# Clustering biological sequences with dynamic sequence similarity threshold

**DOI:** 10.1186/s12859-022-04643-9

**Published:** 2022-03-30

**Authors:** Jimmy Ka Ho Chiu, Rick Twee-Hee Ong

**Affiliations:** Saw Swee Hock School of Public Health, National University of Singapore and National University Health System, Singapore, 117549 Singapore

**Keywords:** Sequence clustering, Graph clustering, Homologous sequences, Metagenomics

## Abstract

**Background:**

Biological sequence clustering is a complicated data clustering problem owing to the high computation costs incurred for pairwise sequence distance calculations through sequence alignments, as well as difficulties in determining parameters for deriving robust clusters. While current approaches are successful in reducing the number of sequence alignments performed, the generated clusters are based on a single sequence identity threshold applied to every cluster. Poor choices of this identity threshold would thus lead to low quality clusters. There is however little support provided to users in selecting thresholds that are well matched with the input sequences.

**Results:**

We present a novel sequence clustering approach called ALFATClust that exploits rapid pairwise alignment-free sequence distance calculations and community detection in graph for clusters generation. Instead of a single threshold applied to every generated cluster, ALFATClust is capable of dynamically determining the cut-off threshold for each individual cluster by considering both cluster separation and intra-cluster sequence similarity. Benchmarking analysis shows that ALFATClust generally outperforms existing approaches by simultaneously maintaining cluster robustness and substantial cluster separation for the benchmark datasets. The software also provides an evaluation report for verifying the quality of the non-singleton clusters obtained.

**Conclusions:**

ALFATClust is able to generate sequence clusters having high intra-cluster sequence similarity and substantial separation between clusters without having users to decide precise similarity cut-off thresholds.

**Supplementary Information:**

The online version contains supplementary material available at 10.1186/s12859-022-04643-9.

## Background

Sequence clustering refers to the process of grouping together similar biological sequences such that only homologous sequences are expected to appear in each sequence cluster. It is particularly useful for identifying various sets of potentially homologous candidates from unknown sequences for further analysis or annotation, as well as aggregating sequencing reads for reference genes abundance estimation in metagenomic samples. Sequence clustering could be considered within the problem domain of general data clustering and are usually resolved using unsupervised learning techniques. Early approaches exploit agglomerative hierarchical clustering [1] to cluster sequences with either single linkage (e.g. BlastClust [2] and GeneRAGE [3]) or average linkage (methods proposed by Loewenstein et al. [4] and Uchiyama [5]) metrics. Partitional clustering, especially K-means clustering [6, 7], was another popular method used to derive sequence clusters (customized K-means approaches by Ashlock et al. [8] and Kelarev et al. [9]). All these approaches require a pairwise sequence distance matrix to be computed. High computation costs are therefore incurred due to the O(*N*^2^) pairwise sequence alignments (such as BLAST alignments [10]) required for *N* sequences. The size of the distance matrix (*N *× *N*) also creates a scalability problem in terms of space complexity. Moreover, clustering results are often sensitive to user-specified clustering parameters. For example, the K-means algorithm would require the number of final clusters *K* to be specified upfront. In order to determine the optimal parameter values for any given set of sequences, the clustering process would need to be performed iteratively, each with a different set of parameter values. After each iteration, an internal validation index [11] such as the silhouette coefficient [12] is calculated from the pairwise sequence distance matrix for the generated output clusters. The set of parameter values having the best index score is deemed to generate the optimal clusters. However, the number of sequences in each cluster is expected to vary substantially [13], with some clusters having hundreds of sequences while some having only a few or even just a single sequence, therefore making it difficult to efficiently estimate the clustering parameters by assuming similar size or density for all clusters.

Many recent sequence clustering approaches [14–18] therefore aim at minimizing both the number of pairwise alignments performed and the space complexity (i.e. the sequence distance matrix is no longer required), as well as defining the cluster cut-off through a biologically comprehensible parameter known as the sequence identity threshold *T*. The value of *T* ranges from 0 (complete mismatch) to 1 (identical sequences) and is usually selected based on users’ domain knowledge or a widely accepted value within the domain. It thus reflects that the higher value of the threshold, the more similar would be the sequences in each derived cluster. Such approaches are primarily greedy algorithms that assign a target sequence to an existing cluster when the pairwise sequence identity of this target sequence with the representative center sequence of this cluster is at least *T*, or otherwise creates a new cluster of size = 1 with this target sequence as its representative center sequence. Techniques such as short word filtering [19, 20] can be applied to avoid the computational expensive procedure of aligning a target sequence with the representative center sequence if their pairwise sequence identity is likely below *T*. The greedy algorithms are also very space efficient because they only consider the sequence identities between the target sequence and all existing representative center sequences. The clustering results can however be affected by the choices of the representative center sequences for the clusters, which are determined by the order of the sequences assessed. MeShClust [21] therefore addresses this limitation by reselecting the representative center sequence from all sequences in the cluster after a new sequence has been added using the mean-shift algorithm [22]. The representative center sequences for all clusters are also reselected again by mean-shift once all sequences have been clustered, and two clusters are merged when the pairwise sequence identity between their representative center sequences is equal to or greater than *T*.

In contrast, MMseqs2 [23] models each sequence as a unique graph vertex, and two vertices are connected by an edge when the pairwise alignment of their underlying sequences satisfies particular criteria including significance, sequence coverage, and *T*. Sequence clusters are then obtained through a graph clustering approach. In addition, the sequence clustering tool Linclust [24] can be run as a pre-processing step to divide the sequences into intermediate clusters for individual graph clustering in each intermediate cluster for scalability. For computational efficiencies, MMseqs2 replaces the exact alignment process between sequences with rapid approximations. The speedup techniques utilized by the different algorithms are summarized in Additional file 1: Table S1. Although the sequence identity threshold *T* is comprehensible to most users, a poorly chosen value could generate clusters substantially different from the true biological clusters [21]. The most common scenario is that the value of *T* is set too high and hence some homologous sequences are assigned to different clusters, but these clusters are hardly identifiable given the large number of sequences or insufficient annotation. Parameter optimization with internal validation index is also not feasible for these approaches due to the absence of the complete distance matrix.

We therefore develop a novel sequence clustering method that dynamically adjusts the cut-off thresholds for individual clusters. It first estimates a complete pairwise sequence distance matrix using an alignment-free approach to avoid performing the traditional slower sequence alignments. This distance matrix is then used to derive all the edge weights for a graph, in which each sequence is represented by a vertex, and the edge weight for an edge denotes the pairwise similarity between the pair of sequences associated with. Sequence clustering is now performed via an iterative graph clustering in which each vertex is regarded as a singleton graph cluster (a singleton graph cluster consists of only one vertex) initially. Each iteration begins with identifying potential clusters to be merged. A cluster separation cut-off threshold then determines which of them can be merged into a single cluster without introducing possible outliers. When a cluster is prohibited from expansion, it is deemed well separated from its neighbours. In contrast to the sequence identity threshold *T*, the cluster separation cut-off threshold is a dynamic threshold because it is partially determined by the clusters considered. We thus name this sequence clustering approach as Alignment-Free Adaptive Threshold Clustering, or ALFATClust in short. ALFATClust is implemented as a publicly available tool, which also provides an user option to evaluate the non-singleton clusters in terms of sequence identity through sequence alignment.

The remaining sections of this manuscript are organized as follows: The “Methods” section first introduces the sequence distance calculation approach and its estimation parameters to be used in ALFATClust, and then gives an overview of the ALFATClust algorithm. Its core steps such as the binning process to derive the graph clusters and the graph contraction are illustrated afterwards. Details of the scalability enhancement and the optional sequence cluster evaluation report are also provided. In the “Results” section, we assess both clustering and time performances of ALFATClust with other sequence clustering tools using the benchmark datasets. We then elaborate on the advantages and limitations of ALFATClust in the “Discussion” section.

## Methods

### Pairwise sequence distance using alignment-free approach

Mash [25] and Dashing [26] are two rapid alignment-free sequence distance approaches that require k-mer size and sketch size to be specified as input parameters. We therefore performed experiments to assess the distance calculation method to be implemented in ALFATClust and also determine the relevant input parameters. The experimental details can be found in Additional file 1 provided. Results (Additional file 1: Figures S1–S5) indicate that Mash is better due to the higher correlation between Mash distance and the sequence distance obtained by alignment approach. In addition, from Additional file 1: Figures S1 and S3, the optimal Mash k-mer size for gene sequences is 17, or lower (e.g. 13) when some sequences are very short. For complete protein sequences, the optimal k-mer size is 9 (Additional file 1: Figure S5). The optimal sketch size is 2000 for both gene and protein sequences. Since Mash distance *d* ranges from 0 to 1, the corresponding sequence similarity can be easily calculated as 1 − *d*.

### Overview of ALFATClust algorithm

The ALFATClust algorithm consists of four components:

1. Mash [25] distance calculation for constructing a graph to model pairwise distances between the input sequences;
2. Leiden algorithm [27] to partition the graph into communities [28] of raw graph clusters;
3. A binning process to bin the vertices in each raw graph cluster into validated graph clusters;
4. A graph contraction step to replace every validated graph cluster by a single vertex.

Suppose a Mash distance matrix *D* is computed for a sequence set *S* such that *d*_*ij*_ of *D* represents the pairwise Mash distance between sequences *s*_*i*_ and *s*_*j*_ in *S*. Since Mash distance is a symmetric distance measure, *d*_*ij*_ = *d*_*ji*_ and both *D* and *W* = 1 − *D* are therefore symmetric matrices. An undirected weighted graph *G* = (*V*, *E*, *W*) is initialized with each vertex *v*_*i*_ ∈ *V* representing a sequence *s*_*i*_ ∈ *S*. Every vertex pair (*v*_*i*_, *v*_*j*_) (*i* < *j*) is connected with an edge *e*_*ij*_ ∈ *E* = {(*v*_*i*_, *v*_*j*_) | *v*_*i*_, *v*_*j*_ ∈ *V*, *i* < *j*}, and the edge weight of *e*_*ij*_ is equal to *w*_*ij*_ of *W*. *w*_*ii*_ = 1 despite the absence of self-loop *e*_*ii*_ in *G*. *w*_*ij*_ therefore denotes the sequence similarity between sequences *s*_*i*_ and *s*_*j*_. Leiden algorithm then partitions the graph into raw graph clusters (communities) that maximize the score calculated using a pre-defined quality function. Given a value of *γ* between 0 and 1, the quality function of the Constant Potts Model (CPM) [29] guarantees that for any vertex *v* in a non-singleton raw graph cluster *R*, the average edge weight of edges connecting between *v* and other vertices in *R* exceeds *γ*. This means the average sequence similarity between the sequences within every non-singleton raw cluster is higher than *γ*. The core parameters of the ALFATClust algorithm thus include a value range [*γ*_*low*_, *γ*_*high*_] (*γ*_*low*_ < *γ*_*high*_) for *γ* and a step size *Δ*, which are used to determine the values of *γ* used in the algorithm:

1. Create graph *G* using Mash distance matrix *D*; assign *γ*_*high*_ to *γ*
2. While *γ* > *γ*_*low *_− *Δ* do:
3. Run Leiden algorithm on *G* using CPM with *γ* to obtain set of raw clusters *C*_*raw*_
4. Initialize *L* with an empty list
5. Assign *C*_*raw*_ to *L* if *γ* = *γ*_*high*_; otherwise bin each raw cluster in *C*_*raw*_ and add the bins to *L*
6. Contract *G* by replacing every graph cluster in *L* by a single vertex and update edges
7. Decrement *γ* by *Δ*
8. Return the sequence clusters represented by *L* derived in the final iteration

The above-mentioned steps would derive graph (sequence) clusters iteratively from *γ* = *γ*_*high*_ to a value determined by *γ*_*low*_. In the *i*-th iteration, it first performs Leiden algorithm using CPM with *γ* = *γ*_*high *_− (*i *− 1)*Δ*, and the raw clusters *C*_*raw*_ become the graph clusters *L* directly in the first iteration. From the second iteration onwards, the vertices in each individual raw cluster are binned (refer to the binning section below) and vertices in the same bin become a validated graph cluster in *L*. After deriving *L* for the current iteration, graph *G* is contracted in a way similar to Uchiyama’s approach [5] that all vertices belonging to a cluster in *L* are replaced by a single vertex. The next iteration, if any, begins with the contracted graph *G*. The graph clusters *L* derived in the final iteration thus become the output sequence clusters. Details of the Leiden algorithm with CPM, binning, and graph contraction are further elaborated below. A detailed description of the ALFATClust algorithm can be found in Additional file 1.

### Leiden algorithm with Constant Potts Model to identify vertices for binning

Community detection [28] refers to the process of partitioning a graph such that vertices in each partition are closely related with each other. While each of these partitions is originally known as a community, we term it as a raw graph cluster or raw cluster in this article. In ALFATClust, community detection is performed using the Leiden algorithm [27] which is an improvement of the Louvain algorithm [30] in both cluster quality and execution time. Briefly, Leiden algorithm starts with each vertex as an individual cluster, and then updates the clusters iteratively to maximize the overall quality score *q*. The calculation of *q* requires a quality function to quantify how closely related the vertices are within each of the raw clusters. For a graph *G* without self-loop, Eq. (1) is the general form of the quality function as defined in CPM [29]:

1 $$q = \mathop \sum \limits_{ i < j} \left( {\alpha_{ij} w_{ij} - \gamma } \right)\delta \left( {\sigma_{i} ,\sigma_{j} } \right)$$

where *α*_*ij*_ belongs to the adjacency matrix *A*. *α*_*ij*_ = 1 for any *i* and *j* because *G* is complete (i.e. an edge exists between any two distinct vertices regardless of edge weight). *σ*_*i*_ refers to the raw cluster for *v*_*i*_, and δ(*σ*_*i*_, *σ*_*j*_) = 1 if *σ*_*i*_ = *σ*_*j*_, i.e., both *v*_*i*_ and *v*_*j*_ belong to the same raw cluster, and zero otherwise. CPM therefore only considers edge weights for edges within a cluster but not those across clusters. The value of *γ*, which is called the resolution parameter, is within the range of the edge weight for *G*, i.e. from 0 to 1. When a vertex exists as a singleton raw cluster it contributes zero score to *q* according to Eq. (1). It follows that the baseline case for CPM is every raw cluster being a singleton cluster and therefore *q* = 0 regardless of the value of *γ*. Given a value of *γ*, for any vertex *v* in a non-singleton raw cluster *R*, the average edge weight of the intra-cluster edges in *R* involving *v* must be greater than *γ* in order to contribute a positive score to *q*. *γ* therefore becomes a lower bound of the average intra-cluster edge weight for all raw clusters. Graph clusters maximizing the value of *q* are regarded as the raw clusters for a particular value of *γ*.

A problem for community detection is the size bias where large communities dominate over small communities [31]. Similar size bias are also observed in CPM where the lower the value of *γ*, the more it favours larger raw cluster size over higher edge weight. For example, suppose graph *G* consists of vertices *v*_*1*_, *v*_*2*_, *v*_*3*_, *v*_*4*_, *v*_*5*_, … with edge weights *w*_*ij*_ in *W*. For *γ* = 0.875, *v*_1_ may form a raw cluster *R* = {*v*_1_, *v*_2_} when *w*_12_ = 0.89, thus giving *q* = 0.89 − 0.875 = 0.15. Meanwhile, *v*_*1*_ cannot form a larger raw cluster *R’* = {*v*_1_, *v*_3_, *v*_4_, *v*_5_} when {*w*_13_, *w*_14_, *w*_15_, *w*_34_, *w*_35_, *w*_45_} = {0.84, 0.86, 0.87, 0.85, 0.87, 0.86}, because *q* for *R’* is equal to (0.84 + 0.86 + 0.87 + 0.85 + 0.87 + 0.86) − 6 × 0.875 = − 0.1. This situation is however reversed for *γ* = 0.85, because *q* becomes 0.05 for *R’* and is now larger than that for *R* (*q* = 0.89 − 0.85 = 0.04). Size bias occurs at *γ* = 0.85 in this example since the highest edge weight *w*_12_ is omitted from *R’* for *v*_*1*_. This suggests, in the presence of size bias, community detection may allocate less similar sequences to the same raw cluster and highly similar sequences to distinct raw clusters. Another problem is that parameter *γ* is still a uniform cluster cut-off threshold analogous to sequence identity threshold *T*. Nevertheless, community detection is still an effective means to identify potential vertices for individual graph clusters provided these shortcomings are addressed. Indeed, ALFATClust minimizes the impact of size bias by deriving the graph clusters iteratively and replacing them with individual vertices through graph contraction. Also, the subsequent binning process facilitates an adaptive cut-off threshold for individual clusters.

### Binning process

The binning cut-off criteria requires calculating the arithmetic mean of the intra-cluster edge weight for every validated graph cluster obtained in the previous iteration. Since a vertex *v*_*i*_ in the contracted graph *G* represents a validated graph cluster, the average intra-cluster edge weight is regarded as the vertex weight of *v*_*i*_ and is denoted as *w*_*ii*_ of *W*. All diagonal elements *w*_*ii*_ of *W* therefore represent the vertex weights for *G* and other elements *w*_*ij*_ (*i* ≠ *j*) denote the edge weights. This does not affect the calculation of Eq. (1) for the community detection process because *G* has no self-loop *e*_*ii*_ and thus *w*_*ii*_ is not associated with any edge. Initially each vertex *v*_*i*_ in *G* is simply a singleton graph cluster, and *w*_*ii*_ = 1 for any singleton cluster represented by *v*_*i*_. For the first iteration (*γ* = *γ*_*high*_), all raw clusters become graph clusters directly without binning. This is because *γ*_*high*_ is supposed to be close to the highest possible edge weight (i.e. 1) so that the raw clusters identified are robust against size bias, and the intra-cluster edge weights are sufficiently large for raw clusters to qualify as graph clusters. The binning process, which is performed individually for each non-singleton raw cluster, begins by sorting its intra-cluster edges in a descending order of edge weight. The two vertices *v*_*i*_ and *v*_*j*_ associated with edge *e*_*ij*_ are added to a vertex list *J* following the sorted edge order, however only the vertex not present in *J* can be added. When both *v*_*i*_ and *v*_*j*_ do not appear in *J*, the one representing the larger underlying graph cluster is added first. The first bin is created using the first vertex in *J*. Starting from the second vertex, the selected vertex *v*_*t*_ is eligible to be assigned to an existing bin *B* satisfying Eq. (2):

2 $$I_{bin} \left( {B,W} \right) - I\left( {B,W,v_{t} } \right) < I\left( {B,W,v_{t} } \right) - \gamma_{low}$$

where

$$\begin{aligned} & I_{bin} \left( {B,W} \right) = \left\{ {\begin{array}{ll} {w_{ii} } \hfill &\quad {if \left| B \right| = 1\; and\; v_{i} \in B} \hfill \\ {\frac{{\mathop \sum \nolimits_{{v_{i} \in B}} \left( {\rho \left( {v_{i} } \right)\left( {\rho \left( {v_{i} } \right) - 1} \right)/2} \right)w_{ii} + \mathop \sum \nolimits_{{v_{i} ,v_{j} \in B,i < j}} \rho \left( {v_{i} } \right)\rho \left( {v_{j} } \right)w_{ij} }}{{\mathop \sum \nolimits_{{v_{i} \in B}} \rho \left( {v_{i} } \right)\left( {\rho \left( {v_{i} } \right) - 1} \right)/2 + \mathop \sum \nolimits_{{v_{i} ,v_{j} \in B,i < j}} \rho \left( {v_{i} } \right)\rho \left( {v_{j} } \right)}}} \hfill &\quad {otherwise} \hfill \\ \end{array} } \right. \\ & I\left( {B,W,v_{t} } \right) = \frac{{\mathop \sum \nolimits_{{v_{i} \in B}} \rho \left( {v_{i} } \right)w_{it} }}{{\mathop \sum \nolimits_{{v_{i} \in B}} \rho \left( {v_{i} } \right)}} \\ \end{aligned}$$

**Fig. 1:**
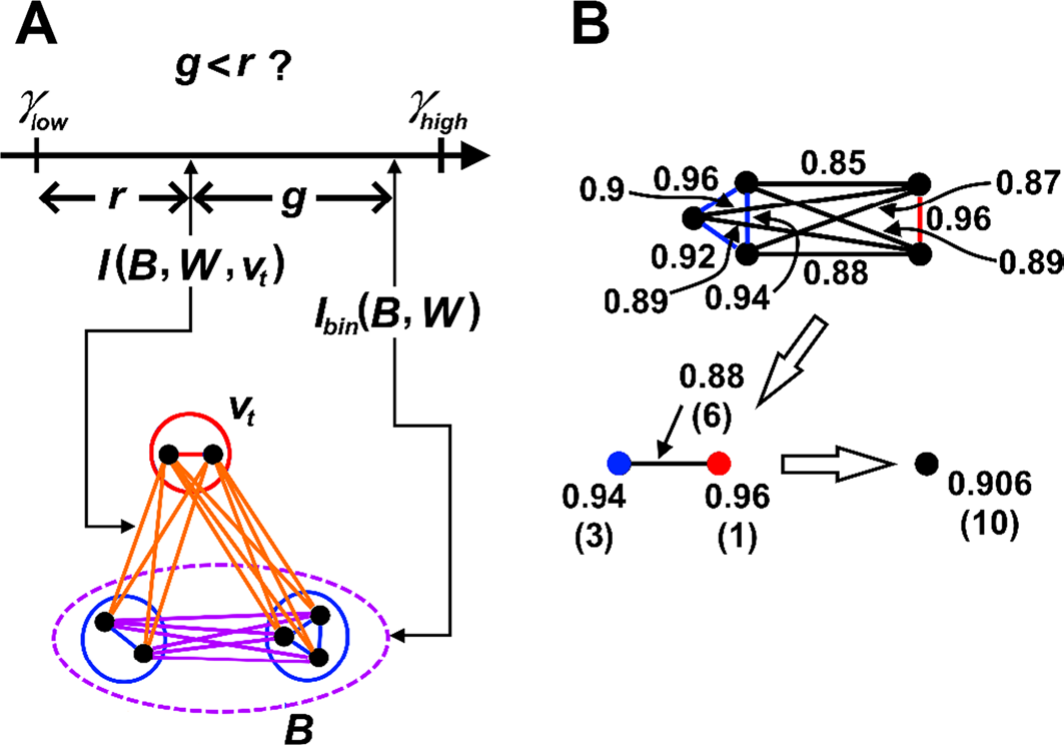
Illustration of the binning process and graph contraction in ALFATClust. **A** Suppose the black dots are the primitive graph vertices. The binning process determines whether a vertex *v*_*t*_ (red circle) representing a graph cluster of two primitive vertices can be assigned to the bin *B* (purple dashed ellipse) consisting of two vertices (blue circles) containing clusters of sizes 2 and 3. *I*_*bin*_(*B*, *W*) is the average edge weight for all edges inter-connecting primitive vertices inside *B* (blue and purple lines) and *I*(*B*, *W*, *v*_*t*_) refers to the average edge weight for all edges (orange lines) connecting *v*_*t*_ and *B*. The cluster separation *g* = *I*_*bin*_(*B*, *W*) − *I*(*B*, *W*, *v*_*t*_) and the cluster cut-off threshold *r* = *I*(*B*, *W*, *v*_*t*_) − *γ*_*low*_. According to Eq. (2), *v*_*t*_ can be assigned to *B* when *g* is less than *r*. **B** The graph contraction shrinks two clusters (primitive vertices connected by either blue or red edges) into two vertices (blue and red dots respectively). The intra-cluster (blue and red) and inter-cluster (black) edge weights are averaged to become vertex (blue and red) weights and collapsed edge (black) weights respectively. The value in the parentheses denotes the number of underlying actual edges. These two vertices can be further collapsed into a single vertex (black)

*ρ*(*v*_*i*_) returns the underlying graph cluster size of *v*_*i*_ as the total number of primitive vertices (i.e. vertices in initial *G*) belonging to that cluster. In Fig. 1A, *I*_*bin*_(*B*, *W*) denotes the current cluster compactness of *B* in terms of its average intra-bin edge weight. *I*(*B*, *W*, *v*_*t*_) refers to the average edge weight between *v*_*t*_ and vertices in *B*. By assuming the homologous sequences have significantly higher edge weights (lower Mash distances) with each other than with other non-homologous sequences, *v*_*t*_ should not be assigned to *B* when *I*(*B*, *W*, *v*_*t*_) is much smaller than *I*_*bin*_(*B*, *W*), or it might introduce outliers into *B* otherwise. *I*_*bin*_(*B*, *W*) − *I*(*B*, *W*, *v*_*t*_) in Eq. (2) is therefore an estimate for the cluster separation between *B* and *v*_*t*_, and *I*(*B*, *W*, *v*_*t*_) − *γ*_*low*_ is the cut-off threshold determined by both *B* and *v*_*t*_ as well as the pre-defined *γ*_*low*_. Figure 1A illustrates both cluster separation and adaptive cut-off threshold in the graph. *v*_*t*_ is assigned to the bin giving the highest positive score calculated with a scoring function *Q*(*B*, *W*, *v*_*t*_):

3 $$Q\left( {B,W,v_{t} } \right) = 2I\left( {B,W,v_{t} } \right) - I_{bin} \left( {B,W} \right) - \gamma_{low}$$

The inequality in Eq. (2) is satisfied when *Q*(*B*, *W*, *v*_*t*_) > 0. *v*_*t*_ is assigned to a new bin when none of the scores for the existing bins are positive. As a result, every bin derived from a raw cluster becomes a validated graph cluster for the current iteration.

### Graph contraction

After binning all raw clusters, *G* is contracted such that each graph cluster *C*_*i*_ in *G* is now replaced by a new vertex *v’*_*i*_, and its vertex weight *w’*_*ii*_ is equal to the average intra-cluster edge weight of *C*_*i*_. Edges connecting between clusters *C*_*i*_ and *C*_*j*_ are replaced by a single edge connecting *v’*_*i*_ and *v’*_*j*_ in the contracted graph, with its edge weight averaged from the replaced edges. Figure 1B illustrates an example of the graph contraction process. However, graph clusters obtained from the same raw cluster are prohibited from appearing together in any raw cluster in subsequent iterations. This is achieved by assigning a large negative edge weight (–|*V’*|^2^ where *V’* is the vertices of the contracted *G*) to the edges interconnecting them. The next iteration, if any, begins with the contracted graph. The graph contraction therefore preserves the validated graph clusters in subsequent iterations against size bias, provided that *Δ* is sufficiently small.

### Selection of the core parameters in ALFATClust

The value of *γ*_*high*_ is supposed to approach the maximum possible edge weight value (i.e. 1) so that the raw clusters detected in the first iteration are robust against size bias. Most of the actual cut-offs for individual clusters are expected to appear above *γ*_*low*_, which still needs to be set as high as possible to avoid capturing subsequent trivial (but larger) drops. For example, a decrease of edge weight from 0.85 to 0.72 is more significant than the next even larger drop from 0.74 to 0.59. The smaller the value of *Δ* the lesser is the size bias for CPM in an iteration. The minimum value for *γ*_*high*_ is at least 0.95. The value for *Δ* should not exceed 0.025. Both *γ*_*high*_ and *Δ* are usually relatively invariant and the value of *γ*_*low*_ can set between 0.7 and 0.8.

### Scalability improvement

Although ALFATClust computes a full Mash distance matrix for its graph clustering, the matrix can be significantly reduced using a divide-and-conquer approach. A pre-clustering step is performed at the beginning to divide the sequences into multiple sequence partitions. This is achieved by running the highly scalable MMSeqs2 with sequence identity threshold *T* equal to *γ*_*low*_, and each of its output sequence clusters becomes an individual sequence partition. ALFATClust can then run with each partition separately without compromising the overall clustering accuracy. This is because the pairwise sequence similarity between any two sequences in different partitions is below *γ*_*low*_, hence filtering their corresponding vertex pair brings little impact to both CPM scoring community detection and the binning process. ALFATClust runs in pre-clustering mode when either the number of sequences exceeds a pre-defined limit (20,000 sequences by default) or the pre-clustering option is activated by the user.

### Cluster evaluation

ALFATClust provides an optional evaluation of cluster quality in terms of sequence identity. For each non-singleton cluster, the sequence giving the largest sum of intra-cluster edge weight and satisfying the sequence length criteria (i.e. between lower quartile − 1.5 × interquartile range and upper quartile + 1.5 × interquartile range) is selected as the representative center sequence. If no such sequence exists or the cluster consists of only two sequences, then the longest sequence will be selected instead. Pairwise sequence identity (number of matched bases divided by alignment length excluding terminal gaps, refer to Additional file 1 for implementation details) is calculated between the center sequence and every other sequence in the same cluster. The evaluation report includes both mean sequence identity and minimum sequence identity for every non-singleton cluster.

## Results

### Benchmark datasets and setup

Antimicrobial resistance (AMR) gene and protein sequence datasets [32–34] are suitable for evaluating clustering effectiveness due to the presence of many distinct classes of homologous resistance gene sequences, and a wide range of sequence similarities among these sequences. AMR gene sequences are retrieved from various public AMR databases including CARD (version 3.0.7) [32], ResFinder (downloaded on 3th Feb, 2020) [33], and ARG-ANNOT (version 4) [34]. All AMR sequences collected are validated, integrated, annotated into an AMR gene sequence dataset using ARGDIT [35], which further translates this dataset into an AMR protein sequence dataset. Non-AMR plasmid nucleotide sequences are extracted with their original annotation from PLSDB [36] (version 2019_10_07) to create a plasmid nucleotides dataset. Due to its highly variable sequence lengths, it can be used to investigate whether the clustering performance is affected when the sequence length differs substantially. Finally, scalability in terms of the number of sequences and sequence length is examined using viral nucleotide and amino acid sequence datasets (each consisting of ~ 470,000 sequences) retrieved from viruSITE (release 2021.1) [37]. Sequences consisting of ambiguous nucleotide or amino acid, or having their sequence length shorter than the smallest Mash k-mer size benchmarked (13 for gene and 9 for protein) are discarded. In particular, viral nucleotide sequences are split into two separate datasets according to the 10,000 nucleotides criteria. The viral sequence datasets are only used for scalability assessments. Table 1 summarizes the properties of all benchmark datasets used, with further details provided in the “Results” section.

**Table 1:** Details of the benchmark datasets used for evaluation

Dataset	No. of sequences	Sequence length		
		Mean (standard deviation)	Min	Max
AMR genes	4027	939.93 (± 381.98)	162	4359
AMR proteins	3891	312.53 (± 127.90)	53	1452
Plasmid nucleotides	5005	1010.38 (± 1 008.45)	77	9511
Viral nucleotides	478,652	717.09 (± 837.21)	13	9993
Long viral nucleotides	676	14,803.87 (± 12 048.56)	10,002	262,388
Viral amino acids	469,835	242.64 (± 313.29)	9	13,556

The performance of ALFATClust is compared with CD-HIT (version 4.8.1), UCLUST (version 11.0.667), VSEARCH (version 2.14.1), DNACLUST (version 3.7), MeShClust (also MeShClust^2^ [38] for the viral nucleotide dataset), and MMseqs2 (version 12.113e3). The identity thresholds used for these tools range from 0.7 to 0.9 with a step size of 0.05. Any cluster optimization option(s) in the tools are also turned on whenever available except for the viral sequence datasets. The suggested value of *γ*_*low*_ is in the range of 0.7 to 0.8 for most instances; in this benchmark *γ*_*low*_ is set to three different values: 0.7, 0.75, and 0.8. Default values are applied for other parameters: *γ*_*high*_ = 0.95, and *Δ* = 0.025. Also, the default Mash k-mer size (17 for nucleotide sequences and 9 for protein sequences) and sketch size (2000) are used except for both plasmid and viral nucleotide datasets where the k-mer size is set to 13. Execution commands and options for all the tools are provided in Additional file 1. Silhouette scores [12] for the sequence clusters are computed using scikit-learn [39], while the results are plotted using Matplotlib [40]. All the benchmarks are performed on a workstation with a quad-core Intel Xeon W-2102 CPU and 64 GB RAM. Cluster evaluation reports of ALFATClust for the AMR and plasmid sequence datasets are available in Additional files 2, 3, 4, 5, 6, 7, 9, 10, 11. In addition, although the Leiden algorithm involves random vertex and community selection, identical set of clusters is obtained from 50 individual runs for each of the AMR and plasmid datasets using any of the three values of *γ*_*low*_ specified above.

### Sensitivity of clustering parameter ***γ***_***low***_ and cluster robustness

**Fig. 2:**
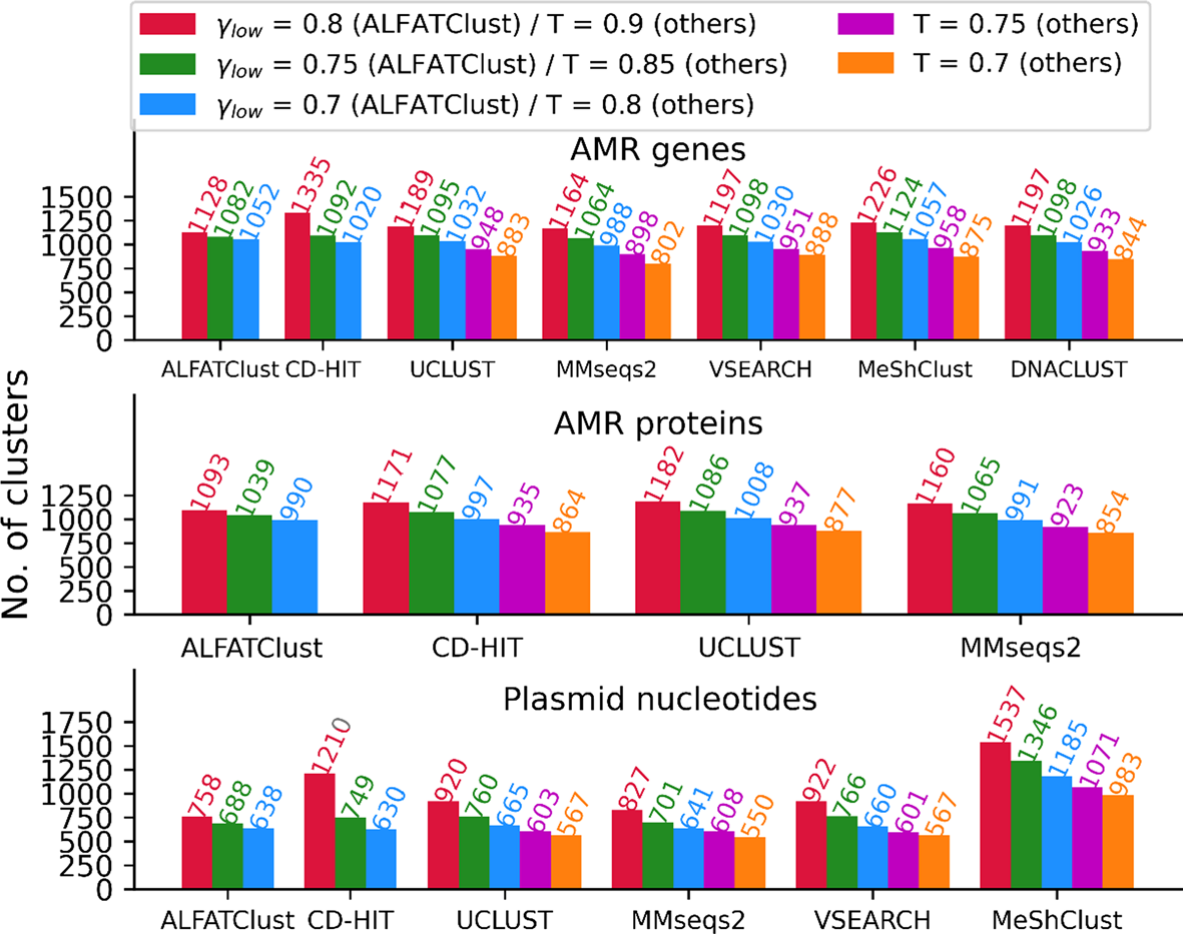
Number of sequence clusters derived by the clustering tools. *γ*_*low*_ for ALFATClust varies from 0.8 down to 0.7 and *T* for others varies from 0.9 down to 0.7, both with a step size of 0.05. The values of *γ*_*low*_ or *T* are distinguished by different colours. Only ALFATClust, CD-HIT, UCLUST, and MMseqs2 can cluster protein sequences. The lowest value of *T* allowed for nucleotide sequences by CD-HIT is 0.8. DNACLUST cannot cluster some plasmid nucleotide sequences that are too short

Figure 2 shows the rate of decrease in number of clusters generated is slower with decreasing *γ*_*low*_ in ALFATClust than decreasing *T* in other clustering approaches, especially for the AMR sequence datasets. One possible explanation is the clustering results being less sensitive to *γ*_*low*_, at least for its suggested value range (0.7–0.8), than *T* in other clustering tools. A further verification is therefore performed by comparing the clusters derived with these values of *γ*_*low*_ and *T* (0.9, 0.85, and 0.8) (Additional file 1: Figures S6–S12). ALFATClust exhibits the highest proportion of identical clusters shared across different *γ*_*low*_. For example, in the AMR gene dataset, the number of identical clusters common across the three values of *γ*_*low*_ is 995 (Additional file 1: Figure S6), which constitute 83.4% of all distinct clusters seen. This proportion is remarkably higher than all other tools such as UCLUST (68.5%, Additional file 1: Figure S8) and MMseqs2 (65.9%, Additional file 1: Figure S9). This better cluster pattern convergence indicates a lower impact of selecting a non-optimal *γ*_*low*_ from the suggested range. Another observation is the uneven distribution of the cluster sizes. When running ALFATClust with *γ*_*low*_ = 0.75, the average number of sequences per cluster and the standard deviation for the AMR gene, AMR protein, and plasmid nucleotides datasets are 3.72 ± 13.77, 3.74 ± 13.98, and 7.27 ± 15.66 respectively. Their cluster sizes are therefore highly varied. Moreover, while there are a few clusters consisting of over 100 sequences in each of these datasets, singleton clusters occupy ~ 70% of all derived clusters for the AMR sequence datasets and ~ 39% for the plasmid dataset. These datasets are thus real examples illustrating the difficulty in searching for suitable clustering parameter value (such as *K* in K-means clustering) evaluated with internal validation index.

**Fig. 3:**
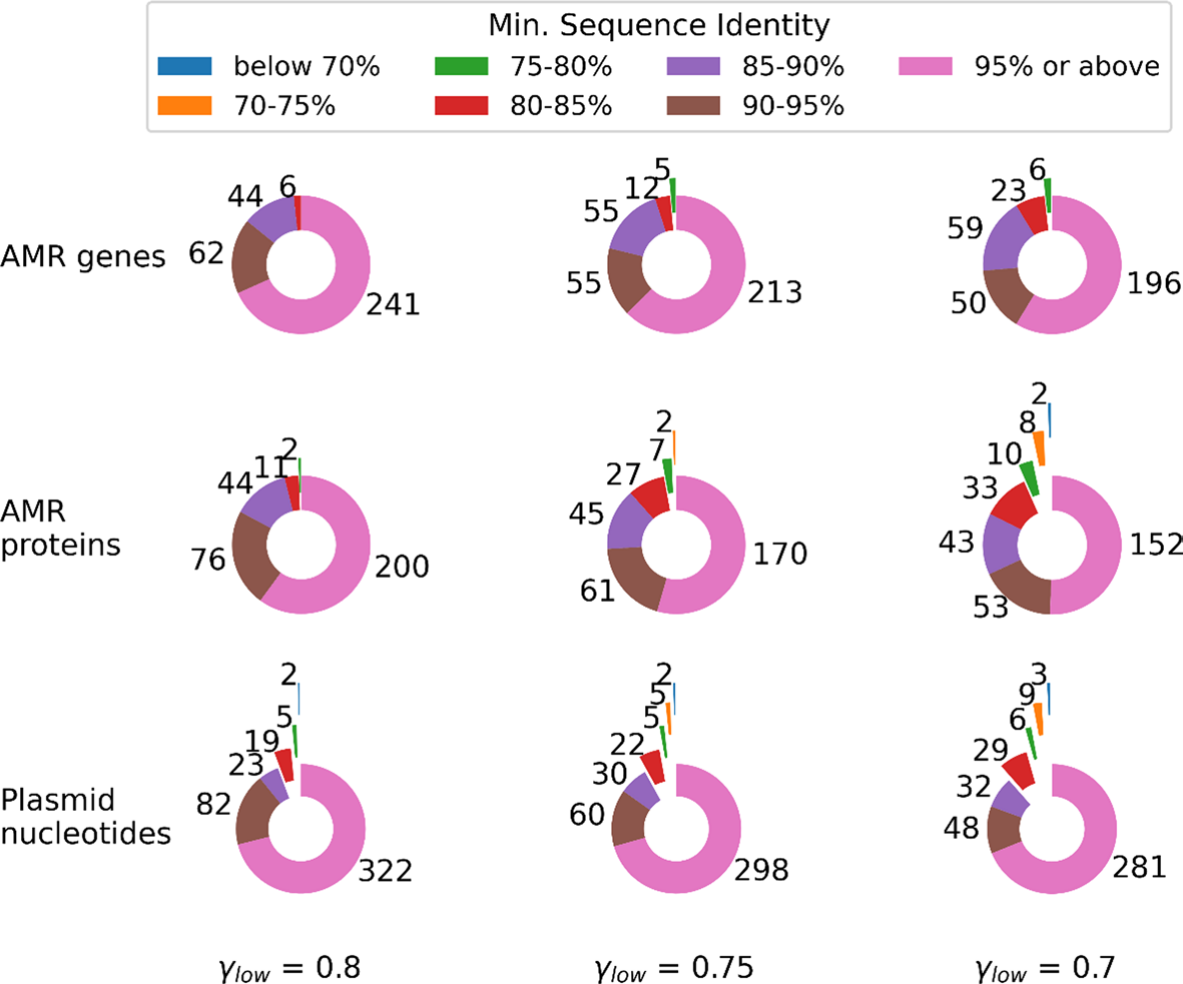
Distribution of non-singleton sequence clusters based on minimum sequence identity. The minimum sequence identity for a sequence cluster is the lowest sequence identity observed between its representative center sequence and another sequence in the same cluster. The value next to the slice indicates the number of clusters belonging to a particular sequence identity category

The distribution of the minimum sequence identities presented in the ALFATClust cluster evaluation reports (available in Additional files 2, 3, 4, 5, 6, 7, 9, 10, 11) is shown in Fig. 3. For *γ*_*low*_ ≥ 0.75, at least 74% of the non-singleton clusters have minimum sequence identity ≥ 0.9 in all three benchmark datasets; and only a few clusters have minimum sequence identity below 0.8. The particularly low sequence identities (< 0.7) for the plasmid nucleotides dataset are partially due to multiple large non-terminal gaps present in the pairwise sequence alignment, such as the one between the cluster center sequence “CP016074.1_rep7a_15_repC(pS0385p1)” and sequence “NC_017335.1_rep7a_18_rep(pS0385p2)” in the same cluster. Another reason is the significantly underestimated Mash distance due to partially overlapping sequence segments. Plasmid nucleotide sequence pair “LT906556.1_IncFII(pCoo)_1_pCoo” and “KX276657.1_IncFIC(FII)_1” is an example demonstrating such partial overlap, with the Mash distance equal to 0.069 (hence the corresponding sequence similarity is 1 − 0.069 = 0.931), while the true sequence identity calculated using alignment is only 0.555. The alignments giving large non-terminal gaps or partial overlap for the above examples are illustrated in Additional file 1. By examining the cluster evaluation reports generated, it is found that for the AMR gene dataset with *γ*_*low*_ = 0.7, the sequence pair having the lowest minimum sequence identity belongs to the same AMR gene family (AMR genes LRA-3 and LRA-9, both under subclass B3 LRA beta-lactamase according to CARD). For the AMR protein dataset with *γ*_*low*_ = 0.7, the sequence pair having the lowest identity score belongs to dihydrofolate reductase (conferring resistance to trimethoprim), although the sequence identity for this pair is quite low (0.675).

### Overall sequence cluster quality benchmark

The overall sequence cluster quality of ALFATClust is compared with other approaches using both external and internal validation indices. Normalized mutual information (NMI) [41] and purity [42] are external validation indices used for this comparison with the AMR sequence datasets. The AMR sequences are manually classified into gene classes created using the gene names (and their synonyms provided by CARD [32]) identified from the AMR gene sequence annotations. Sets of AMR sequence clusters obtained by various approaches at different thresholds are evaluated individually against a set of 827 AMR gene classes consisting of 3 720 AMR sequences (accounting for ~ 92% of all AMR gene sequences, refer to Additional file 8 for the AMR gene classes). For a set of sequence clusters *L* consisting of *N* sequences together, NMI measures the correlation between *L* and the pre-defined gene classes Ω using Eq. (4) below:

4 $$NMI\left( {L,{\Omega }} \right) = \frac{{2 \times I\left( {L;{\Omega }} \right)}}{{H\left( L \right) + H\left( {\Omega } \right)}}$$

where

$$I\left( {L;{\Omega }} \right) = \mathop \sum \limits_{C \in L} \mathop \sum \limits_{{\omega \in {\Omega }}} \frac{{\left| {C \cap \omega } \right|}}{N}\log \left( {\frac{{N\left| {C \cap \omega } \right|}}{\left| C \right|\left| \omega \right|}} \right)$$

and

$$H\left( {\Theta } \right) = - \mathop \sum \limits_{{\theta \in {\Theta }}} \frac{\left| \theta \right|}{N}\log \left( {\frac{\left| \theta \right|}{N}} \right)$$

**Fig. 4:**
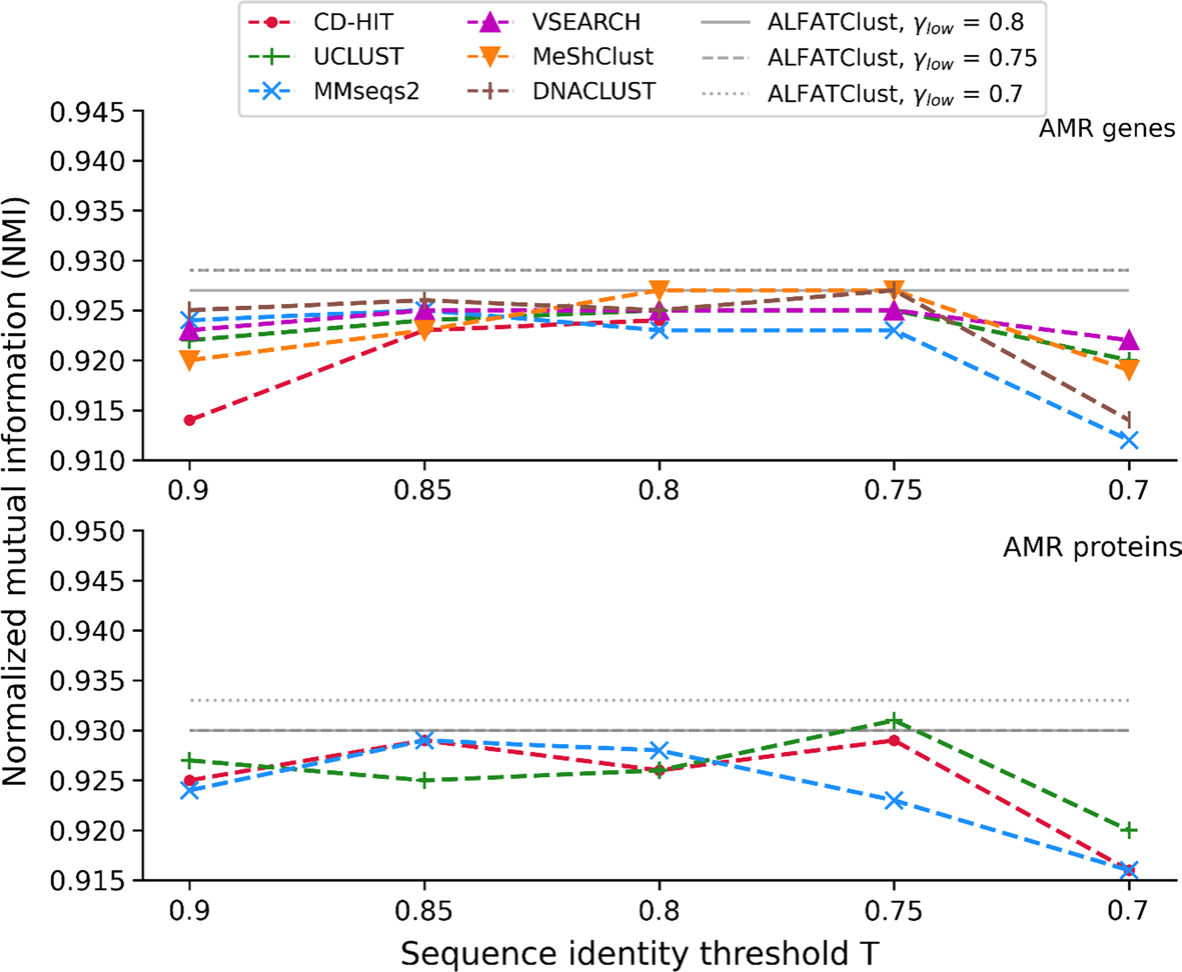
Comparison of NMI between ALFATClust and other clustering tools. *γ*_*low*_ for ALFATClust varies from 0.8 down to 0.7 (solid to dotted lines) and *T* for other clustering tools (x-axis) varies from 0.9 down to 0.7, both with a step size of 0.05. Only ALFATClust, CD-HIT, UCLUST, and MMseqs2 can cluster protein sequences. The lowest value of *T* allowed for nucleotide sequences by CD-HIT is 0.8

The higher the values for NMI (maximum NMI is 1) the better correlation is between the sequence clusters and the gene classes. Figure 4 illustrates how NMI varies with *γ*_*low*_ for ALFATClust as well as *T* for other approaches. The NMI values obtained by ALFATClust are not less than any other tool (Additional file 1: Tables S2 and S3) in the AMR gene dataset, and its NMI at *γ*_*low*_ = 0.7 is the overall highest (0.933) in the AMR protein dataset. Moreover, the NMI value variation for *γ*_*low*_ between 0.7 and 0.8 is relatively small compared to others in both datasets. This is mainly due to the lower sensitivity of the clustering outcomes with *γ*_*low*_ than *T*. Purity is another external index to assess the homogeneity of gene class in the sequence clusters. Higher purity (maximum purity is 1) means the clusters are generally more dominated by sequences in the same gene class. Equation (5) below calculates purity:

5 $$Purity = \frac{1}{N}\mathop \sum \limits_{C \in L} \mathop {\max }\limits_{{\omega \in {\Omega }}} \left| {C \cap \omega } \right|$$

**Fig. 5:**
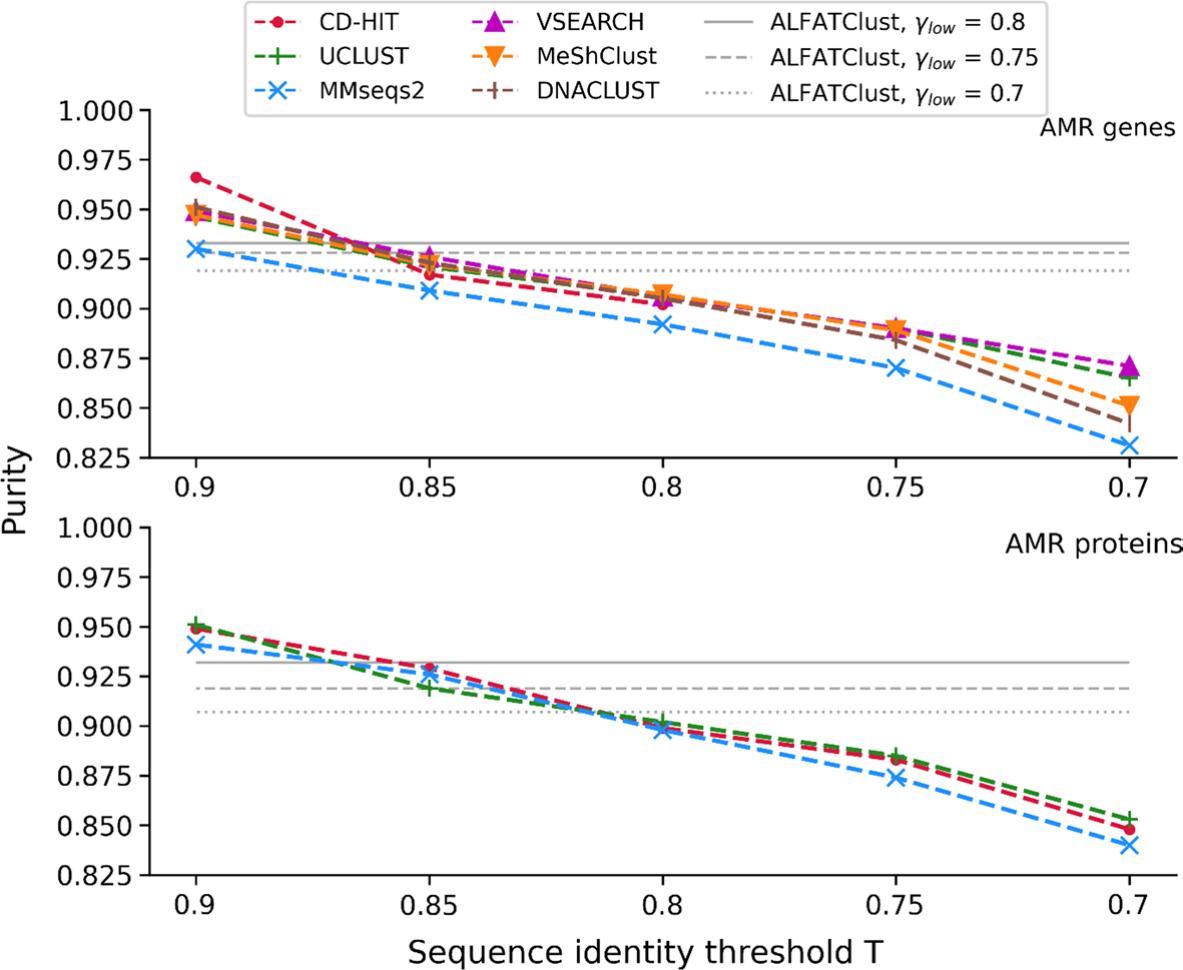
Comparison of purity between ALFATClust and other clustering tools. *γ*_*low*_ for ALFATClust varies from 0.8 down to 0.7 (solid to dotted lines) and *T* for other clustering tools (x-axis) varies from 0.9 down to 0.7, both with a step size of 0.05. Only ALFATClust, CD-HIT, UCLUST, and MMseqs2 can cluster protein sequences. The lowest value of *T* allowed for nucleotide sequences by CD-HIT is 0.8

Figure 5 shows a strictly decreasing trend of purity with decreasing *γ*_*low*_ and *T* for all the clustering approaches benchmarked. Nevertheless, the rate of decrease for ALFATClust is slower than others, and its purity is often close to or even higher than other tools at *T* = 0.85 (exact values for ALFATClust and other methods are shown in Additional file 1: Tables S4 and S5 respectively). The implication is that the cluster expansion in ALFATClust is less aggressive than the greedy algorithms towards lower thresholds. This is the benefit of determining an individual cut-off for each cluster by considering the separation with its neighbour clusters, rather than relying on a single rigid threshold to allocate sequences.

**Fig. 6:**
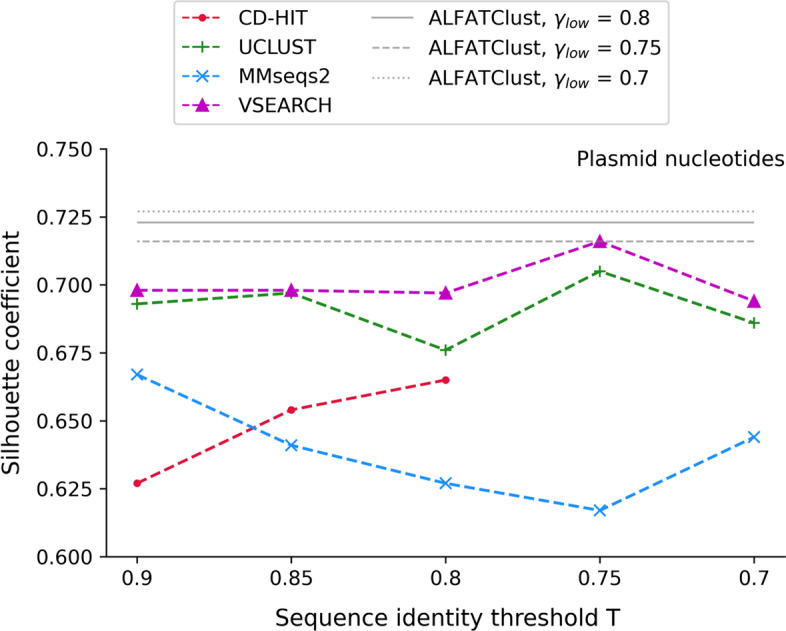
Comparison of silhouette coefficient between ALFATClust and other clustering tools for the plasmid nucleotides dataset. *γ*_*low*_ for ALFATClust varies from 0.8 down to 0.7 (solid to dotted lines) and *T* for other clustering tools (x-axis) varies from 0.9 down to 0.7, both with a step size of 0.05. The lowest value of *T* allowed for nucleotide sequences by CD-HIT is 0.8. DNACLUST cannot cluster some plasmid nucleotide sequences that are too short, and the silhouette coefficients for MeShClust are below zero for the plasmid nucleotides dataset with all values of *T*

Internal validation indices such as silhouette coefficient *c* [12] evaluates the overall cluster separation using the true sequence distance matrix (rather than the Mash distance matrix). *c* is defined as:

6 $$c = \frac{1}{\left| S \right|}\mathop \sum \limits_{s \in S} \frac{{d_{neighbor} \left( s \right) - d_{intra} \left( s \right)}}{{{\text{max}}\left( {d_{neighbor} \left( s \right),d_{intra} \left( s \right)} \right)}}$$

where *d*_*neighbor*_(*s*) measures the separation of sequence *s*, i.e. mean distance between *s* and a sequence in its nearest (called “neighboring”) cluster, and *d*_*intra*_(*s*) measures the cohesion of *s*, i.e. mean distance between *s* and other sequences in the same cluster. *c* ranges from − 1 to 1, and a higher value of *c* indicates a better overall cluster separation. Internal validation indices can be used for cluster quality evaluation when the pre-determined sequence classes are unknown (e.g. the plasmid nucleotides dataset). To compute the true sequence distance matrix for the plasmid nucleotides dataset, exact pairwise sequence identity (same calculation formula as the one used for cluster evaluation in ALFATClust) is calculated for every pair of sequences, and the pairwise distance is given by 1 − sequence identity. Figure 6 shows that while the maximum silhouette coefficient values are attained at distinct values of *T* for different tools, none of them is better than those for ALFATClust at all three values of *γ*_*low*_. By comparing the silhouette coefficients between Additional file 1: Tables S6 and S7, it can be seen that the lowest silhouette coefficient achieved (0.716) by ALFATClust is equal to the highest score obtained with other approaches for the plasmid dataset.

### Scalability performance

The scalability benchmark is performed with respect to the number of sequences using the viral nucleotide and amino acid sequence datasets, and sequence length using the long viral nucleotide dataset. ALFATClust clusters these datasets with *γ*_*low*_ = 0.75 (without the optional cluster evaluation), while others perform clustering with *T* = 0.85. The processing times shown in Table 2 vary substantially among different clustering approaches, ranging from a minute or less to several hours. Both MMSeqs2 and MeShClust runs faster than ALFATClust for the viral nucleotide dataset, but the number of clusters derived by MeShClust is unusually low. UCLUST requires less than a minute to cluster ~ 470,000 viral amino acid sequences, but it cannot process long viral nucleotide sequences due to its software limitation. MMseqs2 as well as the alignment-free ALFATClust and MeShClust^2^ run much faster than other alignment-based tools for long viral nucleotide sequences. In summary, ALFATClust is scalable for a large number of sequences due to the efficient pre-clustering based on MMseqs2, and sequence length through alignment-free sequence distance calculation.

**Table 2:** Scalability benchmark through the viral nucleotide and amino acid sequence datasets

	Datasets					
	Viral nucleotides (≤ 10,000 nts)		Long viral nucleotides (> 10,000 nts)		Viral amino acids^@^	
	No. of clusters	Time (hh:mm:ss)	No. of clusters	Time (hh:mm:ss)	No. of clusters	Time (hh:mm:ss)
ALFATClust^$^	237,276	00:35:41	499	00:00:39	234,451	00:27:23
CD-HIT	N.A.^#^		506	00:16:10	109,515	04:29:06
UCLUST	245,658	07:47:25	N.A.*		243,982	00:00:56
MMseqs2	221,997	00:02:18	428	00:00:15	235,921	00:06:43
VSEARCH	239,728	05:23:11	507	01:48:36		
MeShClust	8713	00:14:53	462	01:14:52		
MeShClust^2^	194,267	03:43:00	571	00:00:10		
DNACLUST	N.A.^^^		N.A.^^^			

^$^ALFATClust runs at *γ*_*low*_ = 0.75, and all other tools run at *T* = 0.85

^#^Terminated after running for 8 h

*Memory limit exceeded for the community (32-bit) version of UCLUST

^Segmentation fault occurs

^@^Only ALFATClust, CD-HIT, UCLUST, and MMseqs2 can process protein sequences

## Discussion

ALFATClust is conceptually similar to hierarchical agglomerative clustering since its algorithm begins with each sequence (vertex) as a singleton graph cluster, and the graph clusters are gradually merged through iterations with decreasing resolution parameter *γ*. The graph contraction at the end of each iteration preserves the integrity of the current graph clusters against the size bias of CPM in subsequent iterations. Moreover, for each raw cluster, vertex sorting prioritizes its vertex pairs in descending order of edge weight (i.e. ascending order of sequence distance) for subsequent binning. Both intra-cluster and inter-cluster edge weight calculations are based on unweighted average linkage as shown in Fig. 1B. Using the CPM formulated in Eq. (1), community detection (Leiden algorithm) acts as a selection process at a particular value of *γ* to identify potential vertices for individual graph clusters. Equation (3) is the scoring function proposed to bin these vertices within each raw cluster to one or more graph clusters. For any existing bin *B*, it considers both the average intra-bin edge weight *I*_*bin*_(*B*, *W*), which is equal to the mean sequence similarity inferring sequence homology, and the proximity between *B* and vertex *v*_*t*_, i.e. *I*(*B*, *W*, *v*_*t*_), with respect to *γ*_*low*_. Note that this scoring function only depends on the constant *γ*_*low*_ but not the variable *γ* in its calculation, hence the binning process is consistent throughout iterations. Although it references a single value of *γ*_*low*_, every bin has its own actual cut-off above *γ*_*low*_. In other words, *γ*_*low*_ is a soft cut-off as opposed to a global hard cut-off like sequence identity threshold *T* or number of clusters *K* [13] to define all output clusters. Individual adaptive cluster cut-offs offer greater flexibility to fit different clusters such that the cut-off value can be lowered for only certain clusters when necessary, i.e. to expand a cluster by including those slightly less similar sequences, if any. The benchmark analysis suggests that the clustering outcomes are less sensitive to different values of *γ*_*low*_ (at least for those within the suggested value range) compared to *T*, therefore reducing the impact of selecting a non-optimal soft cut-off. It is also easier to simultaneously maintain relatively high cluster quality and cluster separation for a wider range of *γ*_*low*_. This is much harder for other algorithms to balance between these two criteria through a uniform cluster cut-off threshold, of which the optimal value is often unknown and difficult to determine. Moreover, ALFATClust is scalable towards sequence length due to the use of alignment-free sequence distance calculation such as Mash.

From the sequence similarity point of view, both the iterative cluster computation from *γ*_*high*_ down to *γ*_*low*_ (provided *Δ* is sufficiently small) accompanied by graph contraction, and the vertex sorting prior to the binning process generally prioritize more similar sequence pairs for clustering. This is particularly important because the linear correlation between the average nucleotide identity (ANI) and the Mash distance *d* decreases with increasing *d* [25], and thus this prioritization allows the sequence pairs to be processed from the most reliable (and small) sequence distances. In addition, since *γ*_*low*_ determines the value of *γ* for the final iteration, most of the pairwise sequence similarity values below *γ*_*low*_ are actually filtered, and so the clustering outcomes are not affected even they deviate substantially from the true sequence identities. Mash distance inaccuracies are mitigated by averaging the edge weights when collapsing the relevant vertices. The proposed ALFATClust algorithm allows pre-clustering to enhance scalability towards number of sequences without disrupting the overall clustering outcomes, because the sequences from distinct partitions are supposed to be dissimilar with each other. Cluster evaluation is also available for users to inspect the quality of the non-singleton sequence clusters, and determine whether the specified value of *γ*_*low*_ is appropriate for the given dataset. In particular, the recommended value for *γ*_*low*_ is 0.7 or above, because the linear correlation between ANI and *d* might become distorted when its value is too low. It is sufficient to set *γ*_*high*_ to 0.95 for most cases, and the step size *Δ* is not larger than 0.025.

Although both ALFATClust and CARNAC-LR [43] are clustering tools based on community detection, they do have fundamental differences. Firstly, CARNAC-LR relies on read alignment (mapping) to determine whether two long reads overlap significantly based on a similarity threshold set in the alignment tool, and an unweighted edge between two vertices (reads) denotes such overlap; ALFATClust creates a complete graph using the alignment-free Mash distances, which are converted to edge weights representing (average) pairwise sequence similarities. Hence, it does not impose any similarity threshold for graph construction. Secondly, CARNAC-LR partitions the graph into *K* cliques or dense subgraphs by minimizing the number of edges between them, and the value of *K* is determined internally; ALFATClust performs sequence clustering based on a user-specified soft cut-off *γ*_*low*_ as explained above. Finally, CARNAC-LR resolves intersecting clusters by identifying spurious read overlaps and cutting the edges accordingly; ALFATClust gradually expands the clusters from the largest (and also the most reliable) edge weights first to minimize the impact of sequence distance approximation errors.

ALFATClust inherits the limitations of Mash distance, particularly distance underestimation for partially overlapping sequences such as the plasmid sequence cases discussed in the “Results” section. Therefore, when the input sequences consist of genome sequence fragments rather than complete gene sequences, users are advised to identify clusters with particularly low sequence identities through the evaluation report to detect potential partial overlaps. Moreover, compared to genome-scale sequences, gene sequences are more sensitive to the specified k-mer size for accurate distance calculation, and this value is shown to be varying between different datasets. It should also be noted that ALFATClust is intended to be used to partition multiple groups of homologous sequences, it is therefore not suitable for tasks such as OTU (operational taxonomic unit) clustering, in which the sequence identity threshold required is strictly 97%.

## Conclusions

Our benchmark demonstrates numerous advantages of ALFATClust over typical threshold-based sequence clustering approaches, including better clustering results for a non-optimal soft cut-off threshold, generally large cluster separation, and scalability with respect to number of sequences and sequence length. It also facilitates cluster quality inspection by providing cluster evaluation. It is suitable for clustering multiple groups of homologous sequences in which the sequence similarity cut-off threshold is often unknown and hard to determine.

## Supplementary Information

**Additional file 1**. Supplementary method and benchmark details, figures, and tables.

**Additional file 2**. ALFATClust cluster evaluation report for the AMR gene sequence dataset (*γ*_low_ = 0.7).

**Additional file 3**. ALFATClust cluster evaluation report for the AMR gene sequence dataset (*γ*_low_ = 0.75).

**Additional file 4**. ALFATClust cluster evaluation report for the AMR gene sequence dataset (*γ*_low_ = 0.8). 

**Additional file 5**. ALFATClust cluster evaluation report for the AMR protein sequence dataset (*γ*_low_ = 0.7).

**Additional file 6**. ALFATClust cluster evaluation report for the AMR protein sequence dataset (*γ*_low_ = 0.75).

**Additional file 7**. ALFATClust cluster evaluation report for the AMR protein sequence dataset (*γ*_low_ = 0.8).

**Additional file 8**. Gene classification for the AMR gene sequence dataset. Used as the background truth for the NMI and purity evaluation.

**Additional file 9**. ALFATClust cluster evaluation report for the plasmid nucleotide sequence dataset (*γ*_low_ = 0.7).

**Additional file 10**. ALFATClust cluster evaluation report for the plasmid nucleotide sequence dataset (*γ*_low_ = 0.75).

**Additional file 11**. ALFATClust cluster evaluation report for the plasmid nucleotide sequence dataset (*γ*_low_ = 0.8).

## Data Availability

ALFATClust is available at https://github.com/phglab/ALFATClust. AMR sequence and plasmid datasets are also available in this package.
