# Supplementary material for: Clustering biological sequences with dynamic sequence similarity threshold
Source: BMC Bioinformatics. 2022 Mar 30;23:108. doi: 10.1186/s12859-022-04643-9 (PMC8969259; doi:10.1186/s12859-022-04643-9)
Supplement: Supplementary file 1 — Additional file 1. Supplementary method and benchmark details, figures, and tables. [file 12859_2022_4643_MOESM1_ESM.pdf]

Clustering biological sequences with dynamic sequence similarity threshold

Jimmy Ka Ho Chiu* and Rick Twee-Hee Ong*

Supplementary material

A. Survey of biological sequence clustering tools

Table S1 below summarizes various threshold-based sequence clustering approaches.

Table S1. Summary of DNA/protein sequence clustering tools included in this benchmark.

Approach	Pairwise identity calculation	Technique for method speedup
CD-HIT (DNA/protein)	Two options:<br>1. b_{match} / l_{short}<br>2. b_{match} / l_{align}	Applies short word (k-mer) filter to avoid aligning query sequence with center sequences that gives pairwise identity < T [1, 2]
UCLUST (DNA/protein)	$b_{match} / (l_{align} - g_{terminal})$	Sorts the center sequences obtained so far in decreasing number of short words shared with the query sequence, then searches from the first center sequence for the one that gives pairwise identity ≥ T when aligning with the query sequence; terminates the search if the first few sequences fail to meet this criteria
DNACLUST (DNA)	$1 - d_{edit} / l_{short}$	Similar to CD-HIT
VSEARCH (DNA)	Four options:<br>1. b_{match} / l_{short}<br>2. b_{match} / l_{align}<br>3. $b_{match} / (l_{align} - g_{terminal})$<br>4. $1 - (b_{mismatch} + g_{open}) / l_{long}$	Similar to UCLUST
MeshClust (DNA)	b_{match} / l_{align}	Predicts sequence identity using a learning approach instead of performing actual sequence alignment
MMseqs2 (DNA/protein)	Three options:<br>1. b_{match} / l_{short}<br>2. b_{match} / l_{long}<br>3. $b_{match} / (l_{align} - g_{terminal})$	Searches for consecutive k-mer matches between any two sequences, and computes the best pairwise ungapped alignments from the search hits, then calculates pairwise gapped alignment score using these ungapped alignments; may opt to run Linclust [3] to divide the sequences into intermediate clusters first for efficient subsequent clustering

b_{match}: number of matched bases/amino acids
$b_{mismatch}$: number of mismatched bases/amino acids
d_{edit}: edit distance = minimum count of insertions, deletions, and substitutions needed to transform one sequence to another
g_{open}: number of gap openings
$g_{terminal}$: length of terminal gaps

l_{short}: length of the shorter sequence
l_{long}: length of the longer sequence
l_{align}: alignment length

B. Selection of alignment-free sequence distance calculation method and its core estimation parameters

We evaluated Mash [4] and Dashing [5] as candidates to be used in ALFATClust for calculating pairwise sequence distances. Both approaches are supposed to approximate the sequence distance as a value between 0 and 1 inclusive. Their sequence distance approximation accuracy is compared with the ground truth using the antimicrobial resistance (AMR) gene and protein datasets, as well as the plasmid nucleotides dataset (refer to the Results section of the manuscript for the dataset details). The ground truth for each dataset is a full pairwise exact sequence distance matrix computed by global pairwise sequence alignment. Based on the global alignment between two sequences, their sequence identity λ is defined as the number of matched bases divided by the alignment length excluding terminal gaps. Since the value of λ ranges from 0 (zero base match) to 1 (identical sequences), the actual pairwise sequence distance is then calculated as $1 - \lambda$ to facilitate direct comparison with Mash or Dashing distance. The scatterplots in Figures S1 – S5 show this comparison for all possible sequence pairs (involving distinct sequences) in different benchmark datasets. In particular, Mash supports both DNA and protein sequences but Dashing only supports DNA sequences. Moreover, the sequence distance is set to 1 if the associated p-value is too high (i.e. the sequence distance calculated is not statistically significant). Various k-mer and sketch size combinations are also compared.

These scatterplots suggest that Mash is more suitable than Dashing not only due to its capability to process protein sequences, but also given its higher correlation with the exact pairwise distance, i.e. by observing a lot more sequence pairs along the diagonal (dashed line). In contrast, Dashing has more substantially underestimated (calculated sequence distance < 0.2 but exact sequence distance ≥ 0.4, indicated by orange points in the figures) sequence pairs than Mash for both AMR and plasmid nucleotide sequences as shown in Supplementary Figures S2 and S4 respectively. The core estimation parameters for both approaches are k-mer size and sketch size. Although the underestimated population decreases with the k-mer size, the Dashing distance becomes greater than 1 for some sequence pairs when the k-mer size is set below 21. When applying Mash for clustering, its estimation parameters are therefore set to meet the following criteria:

1. The total number of underestimated (orange points) and overestimated (exact sequence distance < 0.2 but Mash distance ≥ 0.4, indicated by green points in the figures) sequence pairs is minimized;

2. The non-maximum (< 1) Mash distance reaches ~0.4 for the general sequence pairs. Sequence pairs with Mash distance > 0.4 are often filtered during clustering and so accurate distance calculation beyond 0.4 is unnecessary;

3. Number of random k-mer matches between sequences increases with decreasing k-mer size. Larger k-mer is preferred whenever possible to reduce false hits.

Supplementary Figures S1 and S3 suggest that, based on these criteria, Mash nucleotide k-mer size should be set to ~17 (Mash default is 21) or even lower (e.g. 13) when some of the gene sequences are very short. Default Mash amino acid k-mer size (i.e. 9) appears suitable for complete protein sequences according to Supplementary Figure S5. The sketch size can thus be set to 2 000 for both gene and protein sequences because the overall the correlation does not improve significantly for sketch size over 2 000.

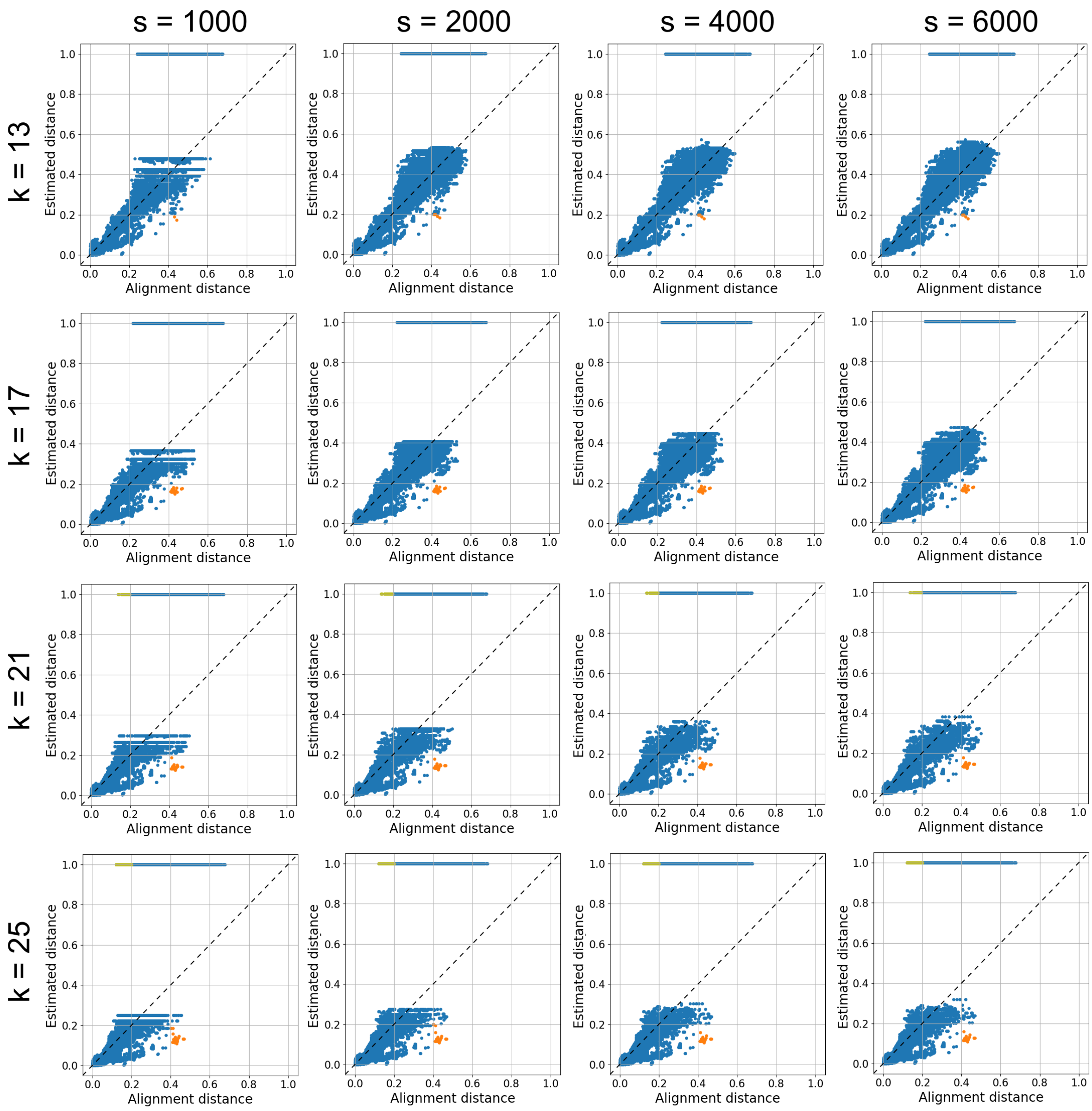

Figure S1. Scatterplots of Mash sequence distance (y-axis) vs actual sequence distance (x-axis) for the AMR gene sequence dataset, with respect to different k-mer size *k* and sketch size *s*. Orange dots correspond to substantially underestimated sequence pairs where Mash distance < 0.2 and actual distance ≥ 0.4; and green dots correspond to substantially overestimated sequence pairs where Mash distance ≥ 0.4 and actual distance < 0.2.

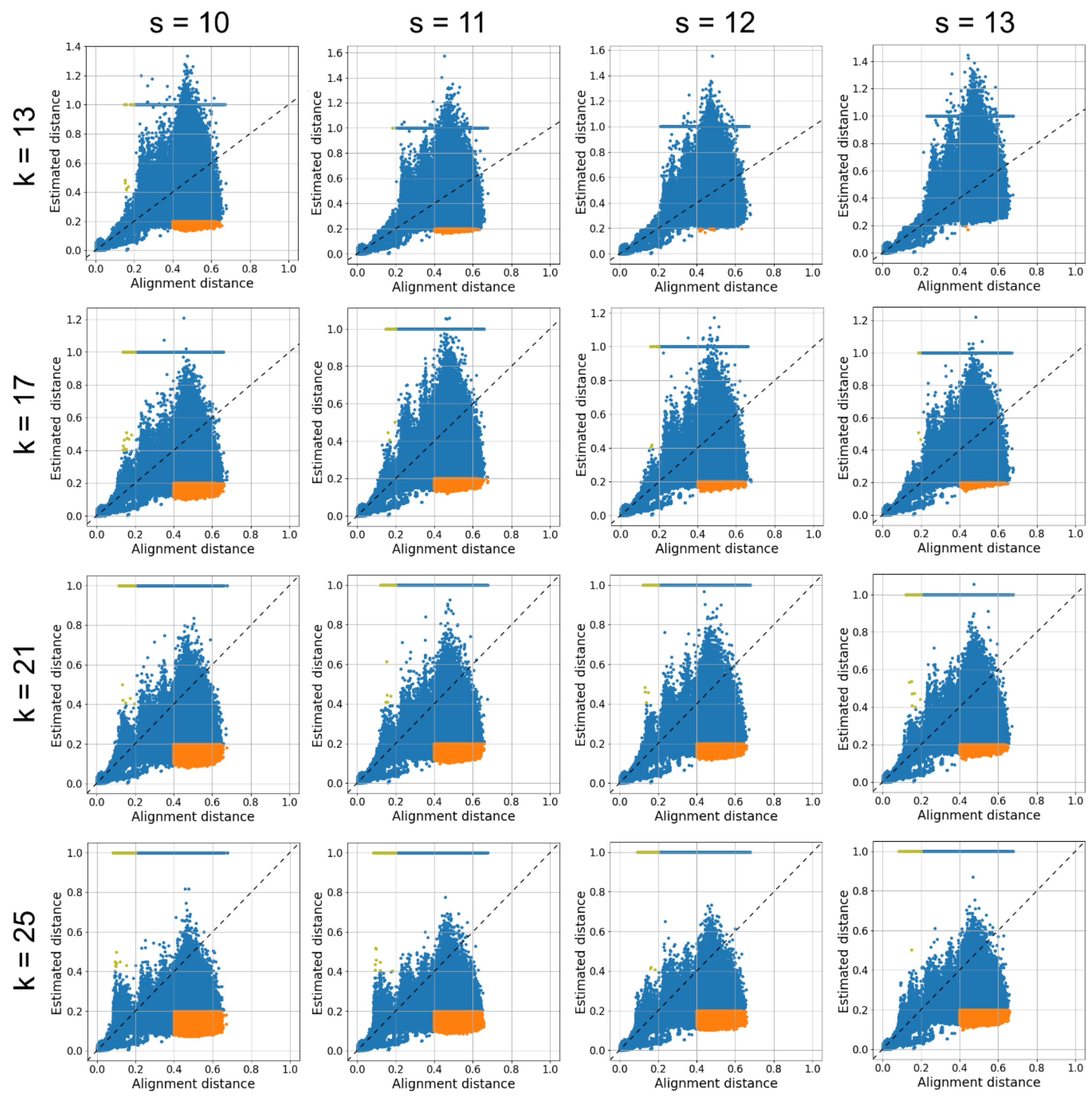

Figure S2. Scatterplots of Dashing sequence distance (y-axis) vs actual sequence distance (x-axis) for the AMR gene sequence dataset, with respect to different k-mer size k and sketch size s. Note that the sketch size for Dashing is 2^s where s is varied from 10 to 13. Orange dots correspond to substantially underestimated sequence pairs where Dashing distance < 0.2 and actual distance ≥ 0.4; and green dots correspond to substantially overestimated sequence pairs where Dashing distance ≥ 0.4 and actual distance < 0.2.

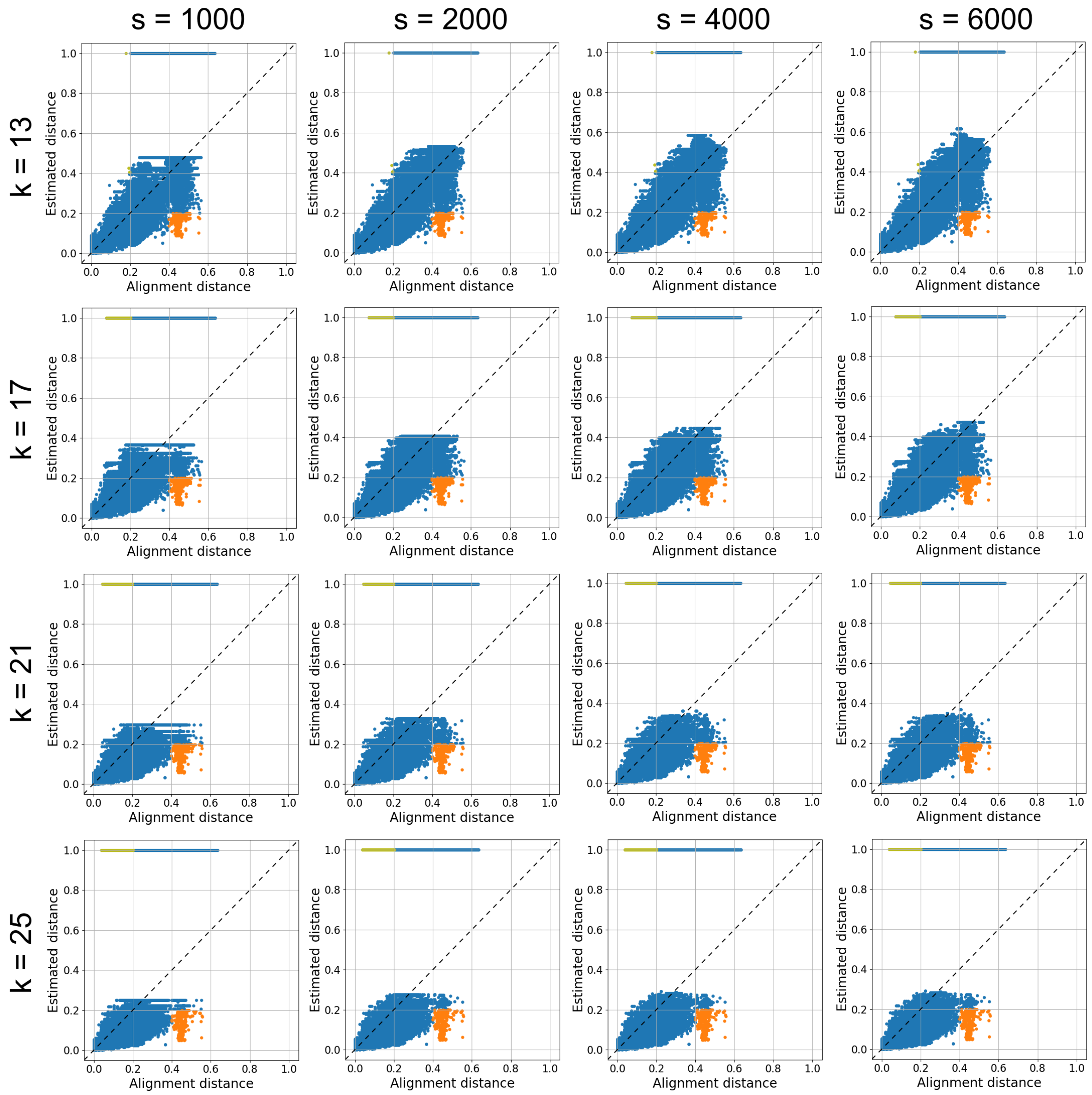

Figure S3. Scatterplots of Mash sequence distance (y-axis) vs actual sequence distance (x-axis) for the plasmid nucleotide sequence dataset, with respect to different k-mer size *k* and sketch size *s*. Orange dots correspond to substantially underestimated sequence pairs where Mash distance < 0.2 and actual distance ≥ 0.4; and green dots correspond to substantially overestimated sequence pairs where Mash distance ≥ 0.4 and actual distance < 0.2.

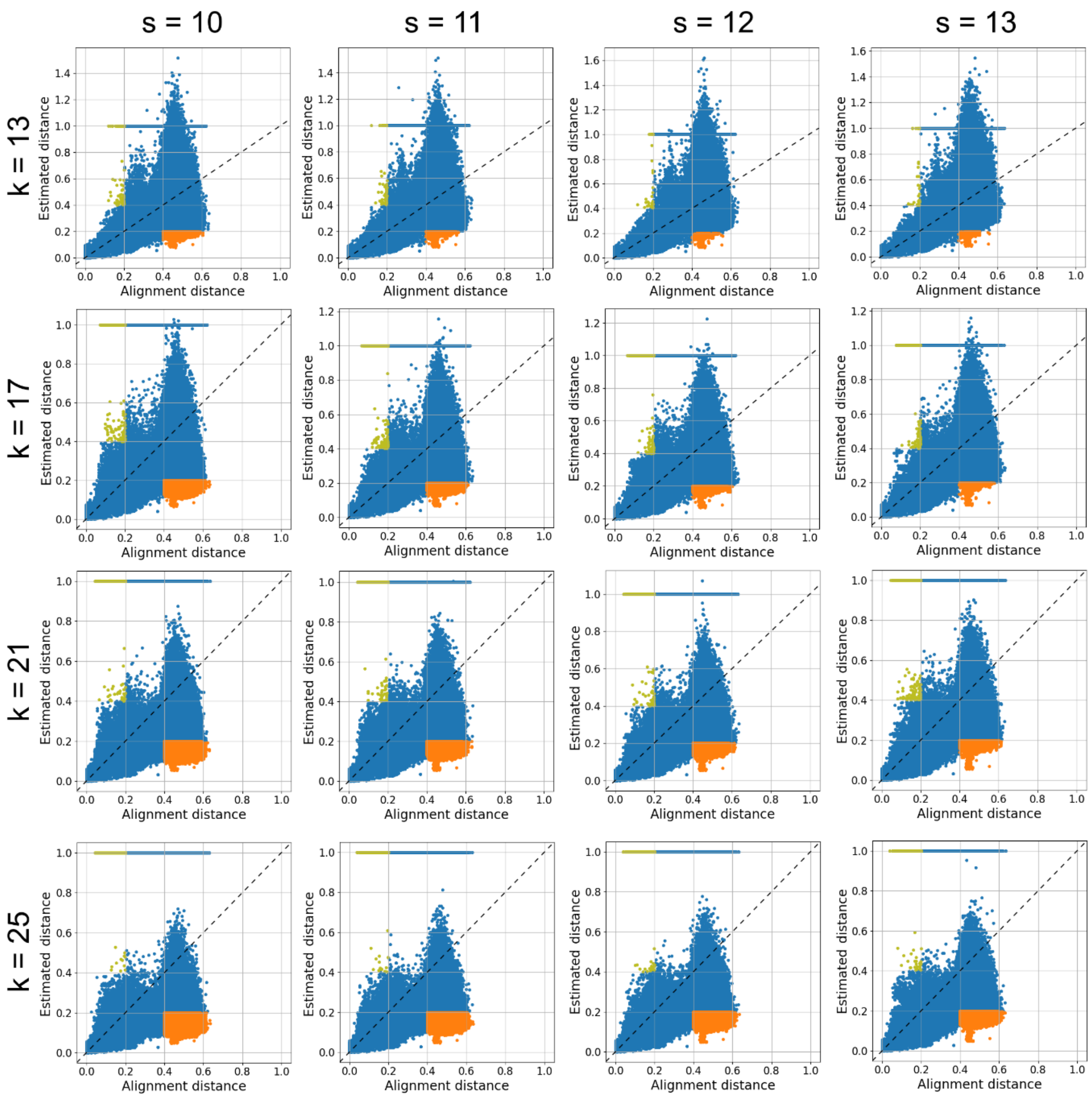

Figure S4. Scatterplots of Dashing sequence distance (y-axis) vs actual sequence distance (x-axis) for the plasmid nucleotide sequence dataset, with respect to different k-mer size k and sketch size s. Note that the sketch size for Dashing is 2^s where s is varied from 10 to 13. Orange dots correspond to substantially underestimated sequence pairs where Dashing distance < 0.2 and actual distance ≥ 0.4; and green dots correspond to substantially overestimated sequence pairs where Dashing distance ≥ 0.4 and actual distance < 0.2.

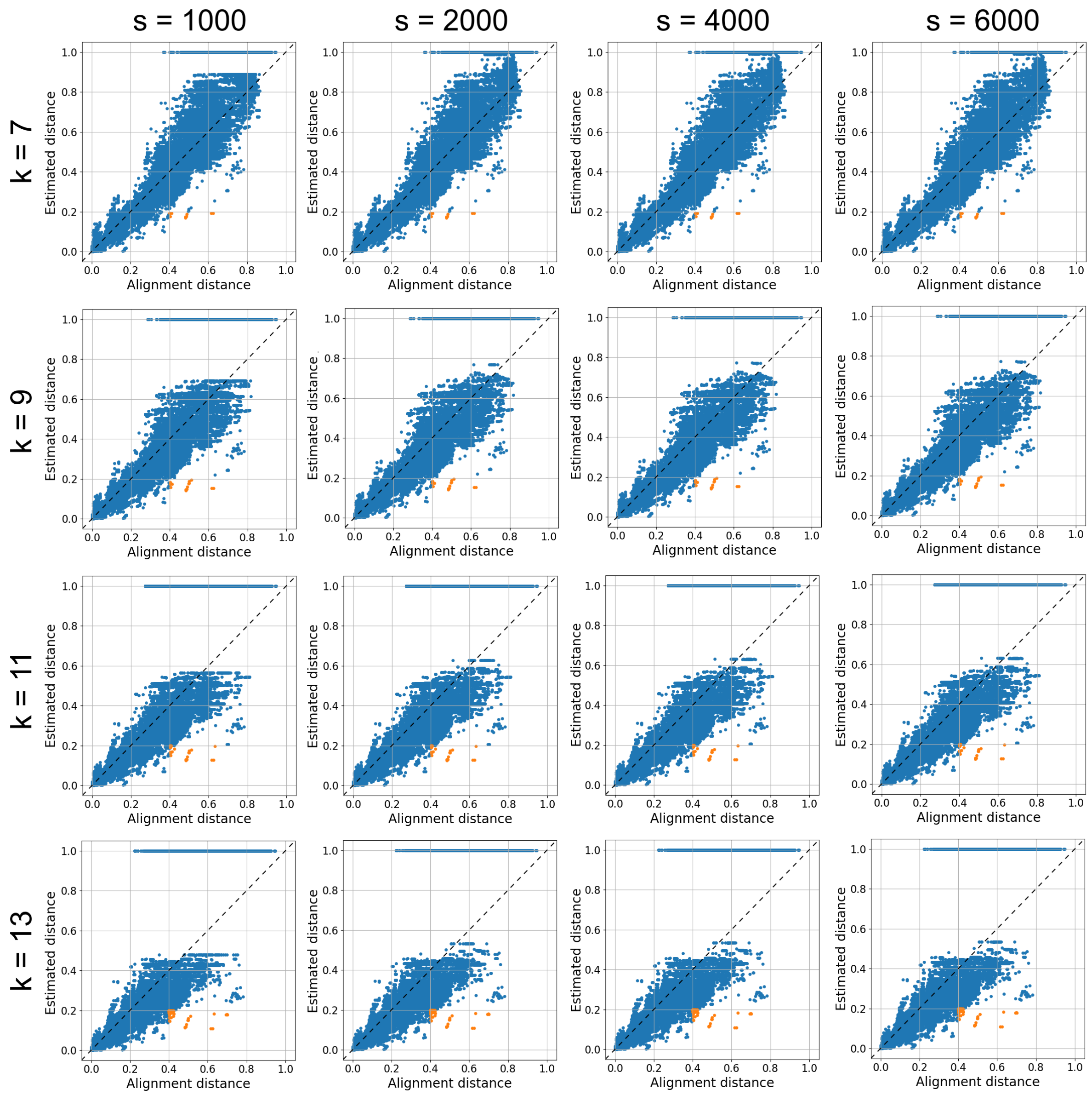

Figure S5. Scatterplots of Mash sequence distance (y-axis) vs actual sequence distance (x-axis) for the AMR protein sequence dataset, with respect to different k-mer size *k* and sketch size *s*. Orange dots correspond to substantially underestimated sequence pairs where Mash distance < 0.2 and actual distance ≥ 0.4.

C. Sequence clustering algorithm

ALFATClust graph clustering algorithm

```
Inputs: D, γ_low, γ_high, Δ   #D is the Mash distance matrix
Output: primitive vertices of cluster in L
bin_raw_cluster(R, W)   #function bin_raw_cluster performs the binning process
  Z ← ∅
  J ← sort vertices in R in descending order of intra-cluster edge weight (edges
      linking between vertices in R) then by number of primitive vertices ρ(v) of each
      vertex v in R
  for each v_t in J
    B ← argmax_{B∈Z} Q(B, W, v_t) s.t. Q(B, W, v_t) > 0   #Q(B, W, v_t) in Equation (3)
    if B = null
      Z ← Z ∪ {{v_t}}
    else
      B ← B ∪ {v_t}
    end if
  end for
  return Z

V ← vertices for D   #start of the main program
E ← {(v_i, v_j) | v_i, v_j ∈ V, i < j}
W ← 1 – D
w_ij ← –|V|^2 ∀ w_ij = 0, i ≠ j   #prevent clustering dissimilar sequences together
G ← (V, E, W)   #w_ij = w_ji
for γ ← γ_high down to γ_low with step size Δ
  L_raw ← leiden(G, γ)   #run Leiden algorithm on G with resolution parameter γ
  X ← ∅
  if γ = γ_high   #no need to bin the raw clusters in the first iteration
    L ← L_raw
  else
    L ← ∅
    for each R in L_raw
      if |R| = 1
        L ← L ∪ {R}
      else
        Z ← bin_raw_cluster(R, W)
        L ← L ∪ Z
        X ← X ∪ {(C_p, C_q) | C_p, C_q ∈ Z, p ≠ q}
      end if
    end for
  end if
  V' ← {v'_i | C_i ∈ L}
  W' ← {w'_ij ← 0 | (v'_i, v'_j) ∈ V' × V'}
  w'_ii ← I_bin(C_i, W) ∀ C_i ∈ L   #I_bin(C_i, W) is defined in Equation (2)
  w'_ij ← (Σ_{v_p∈C_i} Σ_{v_q∈C_j} w_pq ρ(v_p) ρ(v_q)) / (Σ_{v_p∈C_i} Σ_{v_q∈C_j} ρ(v_p) ρ(v_q)) ∀ C_i, C_j ∈ L, i ≠ j, (C_i, C_j) ∉ X
  w'_ij ← –|V'|^2 ∀ w'_ij ≤ 0, i ≠ j
```

40 $V \leftarrow V'$
41 $E \leftarrow \{(v_i, v_j) \mid v_i, v_j \in V, i < j\}$
42 $W \leftarrow W'$
43 $G \leftarrow (V, E, W)$
44 end for
45 return primitive vertices of each cluster C in L

Remarks: $\rho(v)$ in line 38 gives the total number of primitive vertices (i.e. vertices in initial G, line 17) collapsed into v.

D. Pairwise sequence identity calculation in the evaluation report of ALFATClust

Sequence identity between a pair of sequences is calculated based on their best pairwise sequence alignment. When computing the evaluation report in ALFATClust, sequence identity is equal to number of matched bases divided by the alignment length excluding terminal gaps. Although there may be many best pairwise alignments for a sequence pair, not all of them give the highest sequence identity according to a particular definition. Consequently, it would be time consuming to iterate all best alignments to search for the highest value. A heuristic is proposed to reduce the search space. Suppose the two sequences are of different length. No gap penalty is imposed when aligning any terminal region of the longer sequence with gaps. The justification is that the terminal gaps shall appear in the shorter sequence when both sequences are actually similar. The gap penalty is usual for the shorter sequence or both sequences have the same length. Also, the search for the highest sequence identity terminates if no higher value is discovered for five consecutive alignments.

E. Execution of sequence clustering tools

The sequence identity calculation formulas used for benchmark are listed below. Note that for ALFATClust the sequence identity is utilized for cluster evaluation only.

Sequence clustering tool	Sequence identity calculation formula used
ALFATClust	$b_{match} / (l_{align} - g_{terminal})$
CD-HIT [6, 7]	b_{match} / l_{align}
UCLUST [8]	$b_{match} / (l_{align} - g_{terminal})$
VSEARCH [9]	
DNACLUST [10]	$1 - d_{edit} / l_{short}$
MeShClust [11]	b_{match} / l_{align}
MeShClust^2 [12] (viral nucleotide sequence datasets only)	Not applicable (alignment-free)
MMseqs2 [13]	$b_{match} / (l_{align} - g_{terminal})$

b_{match}: number of matched nucleotides/amino acids
d_{edit}: edit distance = minimum count of insertions, deletions, and substitutions needed to transform one sequence to another
$g_{terminal}$: length of terminal gaps
l_{short}: length of the shorter sequence
l_{align}: alignment length

The execution commands for the sequence clustering tools are shown below. Each <…> is an input parameter.

ALFATClust

ALFATClust.is available as a Docker image, which is run as a Docker container to perform the sequence clustering task for all benchmark datasets with the following command:

```
alfatclust -l <lower bound of sequence similarity>
  -i <sequence file path> -o <cluster result file path>
  -e <cluster evaluation file path> -k <k-mer size>
```

Other parameters for ALFATClust are listed in the configuration file *settings.cfg* under the ALFATClust main directory (which is /usr/local/bin/phglab/ALFATClust inside the Docker container).

CD-HIT

The parameter *word size* is adjusted according to CD-HIT user guide:

**Sequence type**	DNA			Protein
**Sequence identity threshold**	0.9	0.85	0.8	0.9 – 0.7
**Word size**	8	6	5	5

AMR and plasmid nucleotides datasets:

```
cd-hit-est -i <sequence file path> -o <cluster result file path>
  -c <sequence identity threshold> -n <word size> -G 0 -aS 0.0001
  -aL 0.0001 -g 1 -d 2000 -T 0
```

AMR protein dataset:

```
cd-hit -i <sequence file path> -o <cluster result file path>
  -c <sequence identity threshold> -n <word size> -G 0 -aS 0.0001
  -aL 0.0001 -g 1 -d 2000 -T 0
```

Parameter “-g 1” is dropped for viral sequence datasets to speed up the clustering process and “-M 0” is specified to allow unlimited memory usage.

Viral gene datasets:

```
cd-hit-est -i <sequence file path> -o <cluster result file path>
  -c <sequence identity threshold> -n <word size> -G 0 -aS 0.0001
  -aL 0.0001 -d 2000 -T 0 -M 0
```

Viral protein dataset:

```
cd-hit -i <sequence file path> -o <cluster result file path>
  -c <sequence identity threshold> -n <word size> -G 0 -aS 0.0001
  -aL 0.0001 -d 2000 -T 0 -M 0
```

UCLUST

```
usearch –cluster_fast <sequence file path>
  -id <sequence identity threshold> -strand both –fulldp
  –uc <cluster result file path>
```

Parameter “-strand both” is used only for nucleotide sequences, and “-fulldp” is dropped for viral sequence datasets to speed up the clustering process.

VSEARCH

```
vsearch --cluster_fast <sequence file path>
  --uc <cluster result file path> --id <sequence identity threshold>
  --strand both
```

DNACLUST

```
dnaclust –i <sequence file path> -s <sequence identity threshold> -l
  -t <number of threads> > <cluster result file path>
```

MeShClust

```
meshclust <sequence file path> --id <sequence identity threshold>
  --output <cluster result file path> --align
  --threads <number of threads>
```

Parameter “--align” is dropped for viral sequence datasets to speed up the clustering process. MeShClust[2] is also used to cluster the viral sequence datasets:

```
meshclust2 <sequence file path> --id <sequence identity threshold>
  --output <cluster result file path> --threads <number of threads>
```

MMseqs2

1. Database creation

   ```
   mmseqs createdb <sequence file path> <database file path>
   ```

2. Clustering

   ```
   mmseqs cluster <database file path>
   <database output cluster file path> <temporary work directory
   path>
   --min-seq-id <sequence identity threshold> -c 0.0001
   ```

3. Result export

   ```
   mmseqs createtsv <database file path> <database file path>
   <database output cluster file path> <output cluster TSV file
   path>
   ```

F. Sequence cluster comparison between different cut-off criteria

Figures S6 – S12 compare the sequence clusters derived with various criteria for each clustering tool benchmarked. The criteria for ALFATClust is the lower bound of resolution parameter γ_{low} (sequence similarity calculated with Mash distance), and is sequence identity threshold T for other clustering tools.

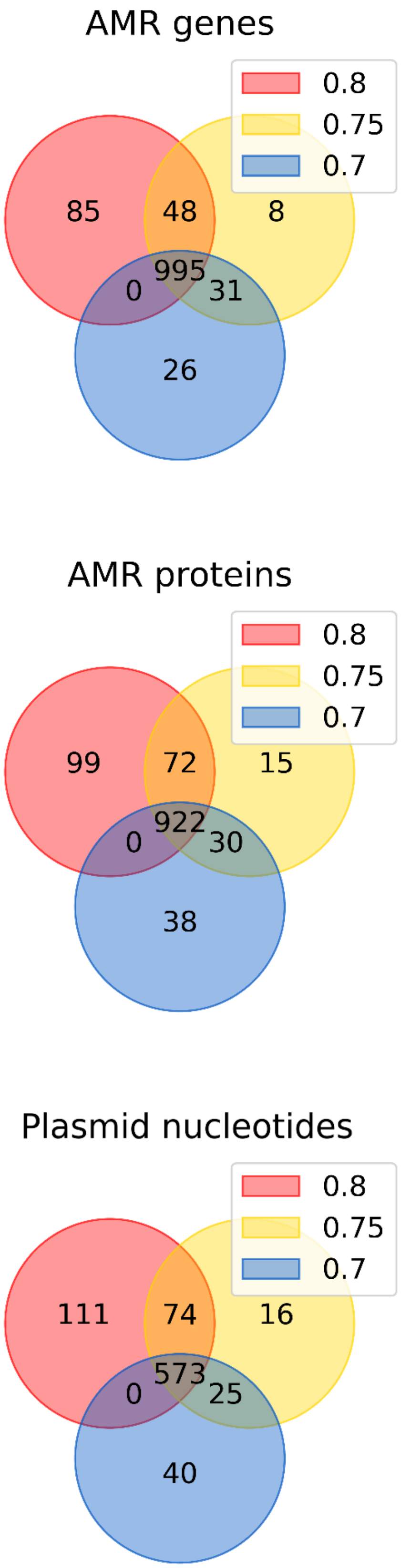

Figure S6. Venn diagrams comparing sequence clusters obtained by ALFATClust with different lower bounds of resolution parameter γ_{low} (0.8, 0.75, and 0.7) for the AMR gene dataset, AMR protein dataset, and plasmid nucleotides dataset (top to bottom).

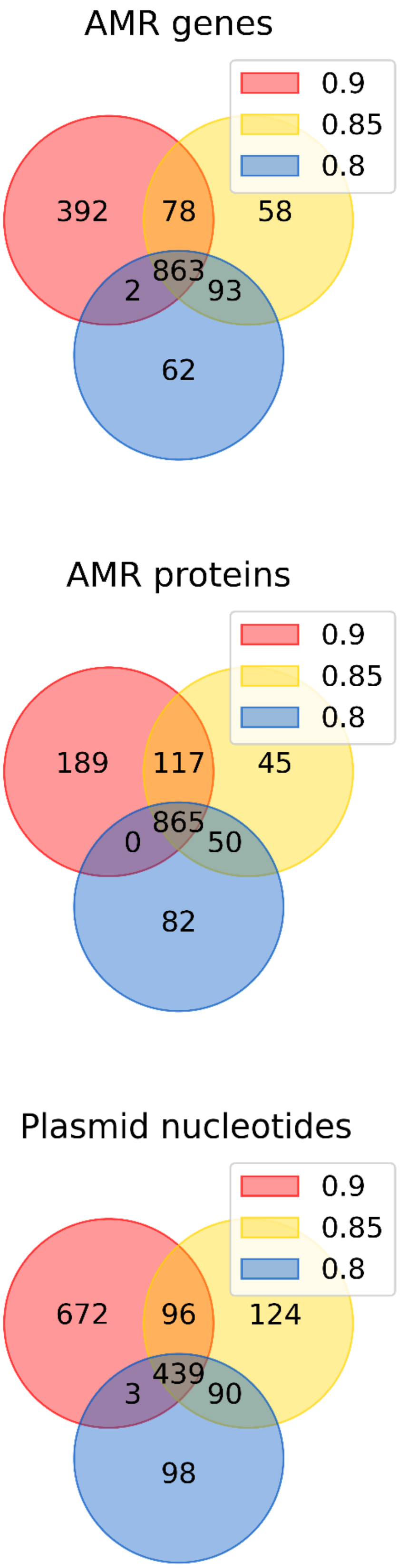

Figure S7. Venn diagrams comparing sequence clusters obtained by CD-HIT with different sequence identity thresholds (0.9, 0.85, and 0.8) for the AMR gene dataset, AMR protein dataset, and plasmid nucleotides dataset (top to bottom).

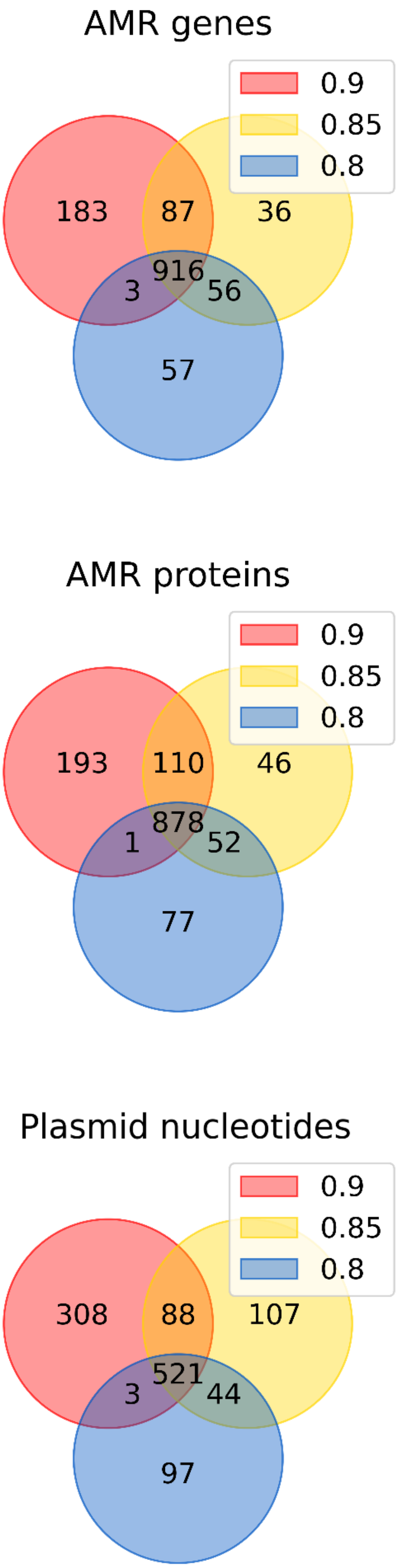

Figure S8. Venn diagrams comparing sequence clusters obtained by UCLUST with different sequence identity thresholds (0.9, 0.85, and 0.8) for the AMR gene dataset, AMR protein dataset, and plasmid nucleotides dataset (top to bottom).

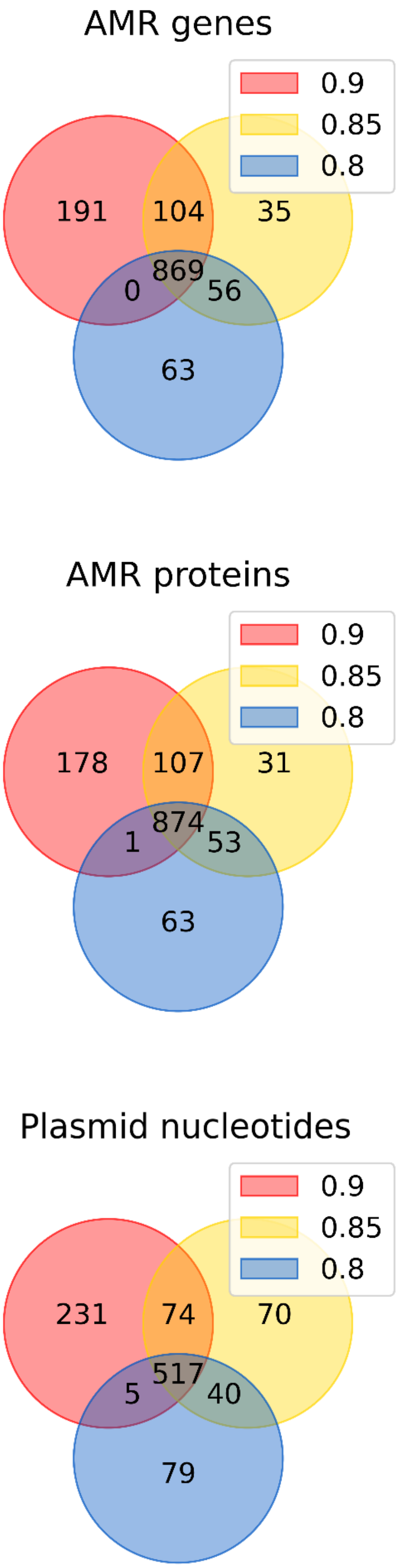

Figure S9. Venn diagrams comparing sequence clusters obtained by MMseqs2 with different sequence identity thresholds (0.9, 0.85, and 0.8) for the AMR gene dataset, AMR protein dataset, and plasmid nucleotides dataset (top to bottom).

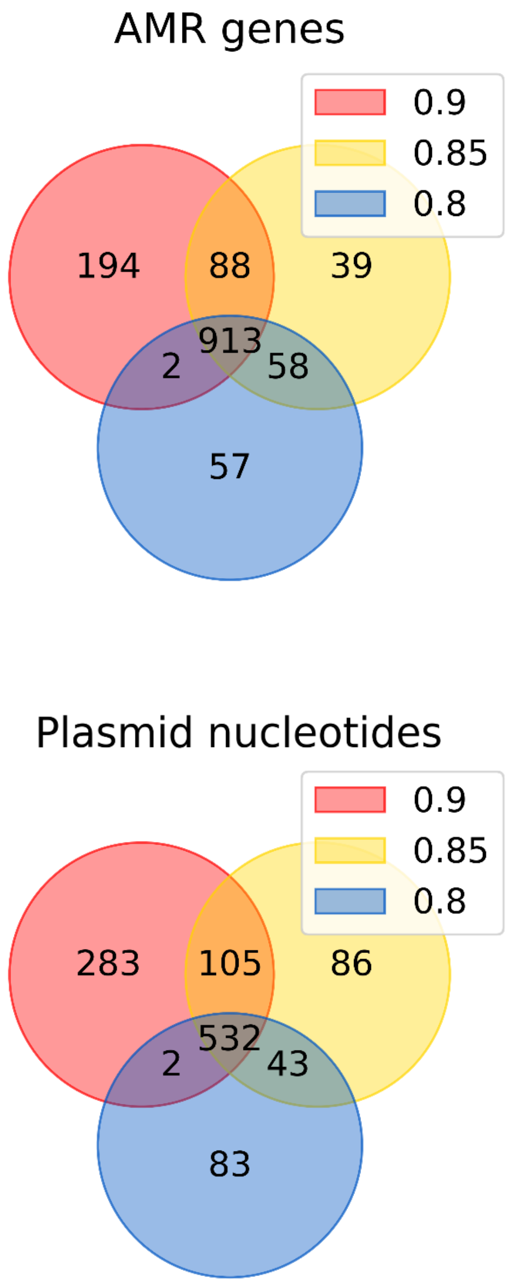

Figure S10. Venn diagrams comparing sequence clusters obtained by VSEARCH with different sequence identity thresholds (0.9, 0.85, and 0.8) for the AMR gene dataset and plasmid nucleotides dataset (top to bottom).

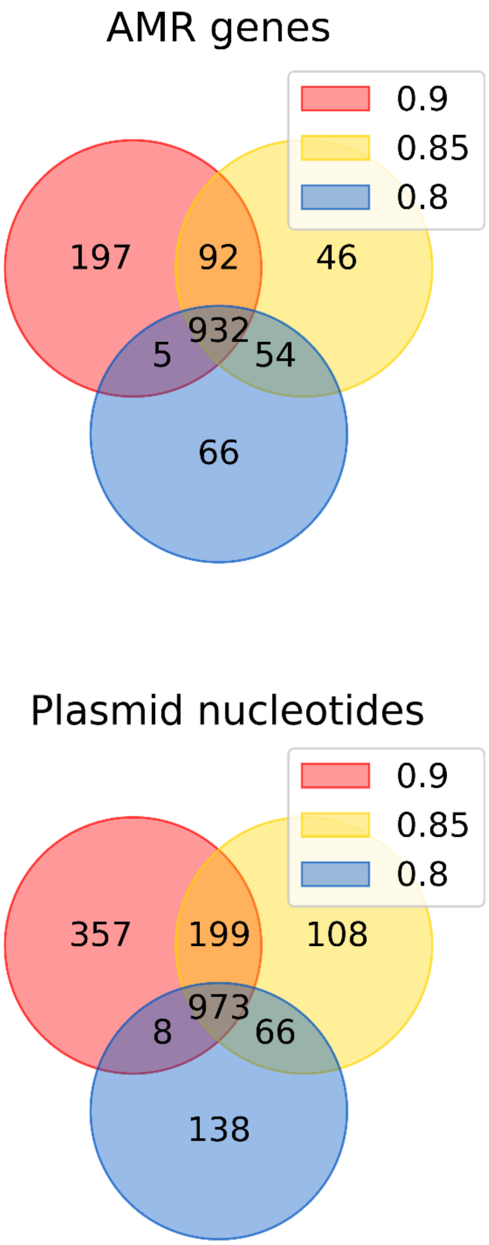

Figure S11. Venn diagrams comparing sequence clusters obtained by MeShClust with different sequence identity thresholds (0.9, 0.85, and 0.8) for the AMR gene dataset and plasmid nucleotides dataset (top to bottom).

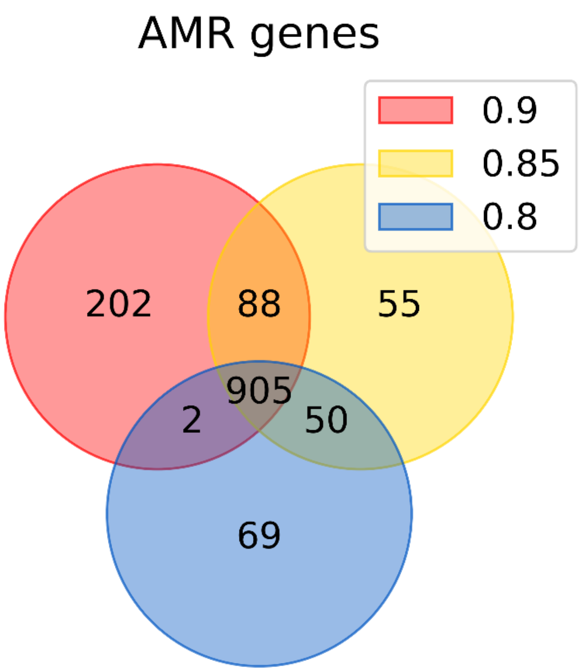

Figure S12. Venn diagrams comparing sequence clusters obtained by DNACLUST with different sequence identity thresholds (0.9, 0.85, and 0.8) for the AMR gene dataset.

G. Explanation of particularly low sequence identity observed in some sequence clusters for the plasmid nucleotides dataset

Since the sequence identity between two biological sequences is calculated from their best pairwise sequence alignment, the result therefore depends on the calculation formula used. In the evaluation report of ALFATClust, sequence identity is equal to the number of matched bases divided by the alignment length excluding terminal gaps. Hence, when the two sequences are of very different lengths, the sequence identity may be quite low even they are highly similar because the large alignment gaps may appear as internal gaps rather than terminal gaps. An example below is between sequences "CP016074.1_rep7a_15_repC(pS0385p1)" (upper, reverse complemented) and "NC_017335.1_rep7a_18_rep(pS0385p2)" (lower) in the plasmid nucleotides dataset. Multiple internal gaps appear near the end of the alignment.

```
------------------------------------------------------------
------------------------------------------------------------
ATGGCAAAAATAACGAAAAATGAGCAAGAAAATTCGGTGAAAAATTACACAAAAAAACCG

------------------------------------------------------------
------------------------------------------------------------
GCGTACTCTAATAGCCGGTTAAACCGACATATTATGTACACCCCCGAACCAAAATTACAC

------------------------------------------------------------
------------------------------------------------------------
TTTGATGCTATGACAATTGTTGGGAATCTTAATAAGAACAATGCTCACAAACTGTCTGAA
```

```
---ATGAGTGTAGAGCCCCAAATAAGACTTTGGGATATTCTTCAAACAAAGTTTAAAGCT
---||||||.|.|.|||.|||||.||||||||||||||..|.|||||.||.|||||||||
TTTATGAGTATTGCGCCACAAATTAGACTTTGGGATATATTACAAACCAAATTTAAAGCT

AAAGCACTTCAAGAAAAAGTTTATATTGAATATGACAAAGTGAAAGCAGATAGTTGGGAT
||.||.||.||||||||||||||.||.||||||||.|||||.|||||||||...||||||
AAGGCTCTACAAGAAAAAGTTTACATCGAATATGATAAAGTAAAAGCAGATGCGTGGGAT

AGACGTAATATGCGTATTGAATTTAATCCAAACAAACTTACACGAGATGAAATGATTTGG
|||||||||||||||.|||||||||||||.||.||||||||.|..||.||||||.|||||
AGACGTAATATGCGTGTTGAATTTAATCCTAATAAACTTACGCATGAAGAAATGCTTTGG

TTAAAACAAAATATAATAAGCTACATGGAAGATGACGGTTTTACAAGATTAGATTTAGCC
|||||||||||.||.||...|||||||||||||||.||||||||||||||||||||.||.
TTAAAACAAAACATCATTGACTACATGGAAGATGATGGTTTTACAAGATTAGATTTGGCT

TTTGATTTTGAAGATGATTTGAGTGACTACTATGCAATGTCTGATAAAGCAGTTAAGAAA
||||||||||||||.||||||||.||.||.|||||.|||.||||||||.|||||||||||
TTTGATTTTGAAGACGATTTGAGCGATTATTATGCGATGACTGATAAATCAGTTAAGAAA

ACTATTTTTTATGGTCGTAATGGTAAGCCAGAAACAAAATATTTTGGCGTGAGAGATAGT
||||||||||||||||||||.|||||.||||||||||||||||||||.||..|.||.|||
ACTATTTTTTATGGTCGTAACGGTAAACCAGAAACAAAATATTTTGGTGTTCGTGACAGT

AATAGATTTATTAGAATTTATAATAAAAAGCAAGAACGTAAAGATAATGCAGATGCTGAA
.||||||||||||||||||||||||||||.|||||||||||.|||||||||||...||||
GATAGATTTATTAGAATTTATAATAAAAAACAAGAACGTAAGGATAATGCAGACATTGAA

GTTATGTCTGAACATTTATGGCGTGTAGAAATCGAACTTAAAAGAGATATGGTGGATTAC
||||||||||||||.|||||||||||||||||.|||.|.||||||||||||||.|||||.
GTTATGTCTGAACACTTATGGCGTGTAGAAATTGAATTAAAAAGAGATATGGTTGATTAT

TGGAATGATTGCTTTAGTGATTTACATATCTTGCAACCAGATTGGAAAACTATCCAACGC
|||||.|||||.||||.||||||||||||.|||.|||||||||||-------||------
TGGAACGATTGTTTTAATGATTTACATATTTTGAAACCAGATTGG-------TC------

ACTGCGGATAGAGCAATAGTTTTTATGTTATTGAGTGATGAAGAAGAATGGGGAAAGCTT
----------------||||||-------------------||||--------||||---
----------------TAGTTT-------------------AGAA--------AAAG---

CACAGAAATTCTAGAACAAAATATAAGAATTTGATAAAAGAAATTTCGCCAGTCGATTTA
----------------------------------|||||||-------|||--------|
----------------------------------TAAAAGA-------CCA--------A

ACGGACTTAATGAAATCGACTTTAAAAGCGAACGAAAAACAATTGCAAAAACAAATCGAT
.|------|||||-------||||.|-----------------|||.||----------|
GC------AATGA-------TTTATA-----------------TGCTAA----------T

TTTTGGCAACATGAATTTAAATTTTGGAAATA
|--------|||--------------------
T--------CAT--------------------
```

Another cause is related to Mash which substantially underestimates the distance between two sequences in which leftmost segment of one sequence overlaps with the rightmost segment of

another sequence, or vice versa. The pairwise sequence alignment below illustrates this partial overlap between sequences “LT906556.1_IncFII(pCoo)_1_pCoo” (upper) and “KX276657.1_IncFIC(FII)_1” (lower, reverse complemented) in the plasmid nucleotides dataset.

```
CACACCATCCTGCACTTACAATGCGCAGAAGGAGTGAGCACAGAAAGAAGTCTTGAACTT
------------------------------------------------------------
------------------------------------------------------------

TTCCGGGCATATAACTATACTCCCCGCATAGCTGAATTGTTGGCTATACGGTTTTTAGAT
------------------------------------------------------------
------------------------------------------------------------

GGCCCCGGTAATCTTTCTCACTCGCCAAAGTTAAAGAAGATTATCGGGGTTTTCACTTTT
-----------------------------------|||||||||||||||||||.|||||
-----------------------------------GAAGATTATCGGGGTTTTCGCTTTT

CTGGCTCCTGTAAATCCACATCAGAACCAGTTCCCTGCCACCTTACGGCGTGGCCAGCCA
||||||||||||||||||||||||||||||||||||||||||||||||||||||||||||
CTGGCTCCTGTAAATCCACATCAGAACCAGTTCCCTGCCACCTTACGGCGTGGCCAGCCA

CAAAATTCCTTAAACGATCAG---------------------------------------
|||||||||||||||||||||---------------------------------------
CAAAATTCCTTAAACGATCAGCAATCTACCACTCACTCCTGAGATAAGCAAGAATGTGAA

------------------------------------------------------------
------------------------------------------------------------
TATTTACGAAGTCGCTCTGCGTTTCAGCTCTGATTCAATCAGTTTTTCAAGCATCTGCGC

------------------------------------------------------------
------------------------------------------------------------
CTGAGTAATACCTTCCTCATCCGCCAGTTCACTCAGCCTGTCTTTTAACCGGGCCTGAAT

------------------------------------------------------------
------------------------------------------------------------
AACCGCATGAAAAGCCTTATGAGTGTTGCTTCTGCGAGCCAGAGAAGCTCTTTGCCTCTC

-------------------
-------------------
TCTGGCCGGAACAGGGTTA
```

The above alignment merely illustrates why Mash returns a very low sequence distance (0.069) between these two sequences, and is often not considered as the best alignment since the overlap region is too short.

H. Normalized mutual information, purity, and silhouette coefficients comparison

The AMR gene sequences in the benchmark datasets are annotated by ARGDIT [14] with the information retrieved from the NCBI nucleotide repository. These sequences are classified based on the AMR gene names and synonyms (according to CARD database v3.1.1 [15]) identified from their annotations (assuming they are correct), and then by pairwise sequence identities when necessary. 3 720 out of 4 027 AMR gene sequences (~92%) are classified into 827 classes, which are also applicable to the AMR protein sequences due to identical annotations. The sequence classification file *expanded_gene_class_seqs.csv* is available together with this supplementary material. The classification results are used to calculate the normalized mutual information (NMI) and purity for sequence cluster evaluation.

Tables S2 and S3 show the NMI of the sequence clusters derived by ALFATClust and other clustering tools respectively for the AMR gene and AMR protein.

Table S2. NMI of sequence clusters derived by ALFATClust for the AMR datasets.

	γ_{low}		
	**0.8**	**0.75**	**0.7**
**AMR genes**	0.927	0.929	0.929
**AMR proteins**	0.93	0.93	0.933

Table S3. NMI of sequence clusters derived by other clustering tools for the AMR datasets.

**AMR genes**					
	T				
	**0.9**	**0.85**	**0.8**	**0.75**	**0.7**
**CD-HIT**	0.914	0.923	0.924	N.A.[#]	N.A.[#]
**UCLUST**	0.922	0.924	0.925	0.925	0.92
**MMseqs2**	0.924	0.925	0.923	0.923	0.912
**VSEARCH**	0.923	0.925	0.925	0.925	0.922
**MeShClust**	0.92	0.923	0.927	0.927	0.919
**DNACLUST**	0.925	0.926	0.925	0.927	0.914
**AMR proteins**[*]					
	T				
	**0.9**	**0.85**	**0.8**	**0.75**	**0.7**
**CD-HIT**	0.925	0.929	0.926	0.929	0.916
**UCLUST**	0.927	0.925	0.926	0.931	0.92
**MMseqs2**	0.924	0.929	0.928	0.923	0.916

[#]: The lowest value of *T* allowed for CD-HIT is 0.8

[*]: Only CD-HIT, UCLUST, and MMseqs2 can cluster protein sequences

Tables S4 and S5 show the purity of the sequence clusters derived by ALFATClust and other clustering tools respectively for the AMR gene and AMR protein.

Table S4. Purity of sequence clusters derived by ALFATClust for the AMR datasets.

	γ_{low}		
	**0.8**	**0.75**	**0.7**
**AMR genes**	0.933	0.928	0.919
**AMR proteins**	0.932	0.919	0.907

Table S5. Purity of sequence clusters derived by other clustering tools for the AMR datasets.

**AMR genes**					
	T				
	**0.9**	**0.85**	**0.8**	**0.75**	**0.7**
**CD-HIT**	0.966	0.917	0.902	N.A.#	N.A.#
**UCLUST**	0.946	0.921	0.906	0.89	0.865
**MMseqs2**	0.93	0.909	0.892	0.87	0.831
**VSEARCH**	0.949	0.926	0.906	0.89	0.871
**MeShClust**	0.947	0.922	0.907	0.889	0.851
**DNACLUST**	0.951	0.923	0.905	0.884	0.842
**AMR proteins***					
	T				
	**0.9**	**0.85**	**0.8**	**0.75**	**0.7**
**CD-HIT**	0.949	0.929	0.899	0.883	0.848
**UCLUST**	0.951	0.919	0.902	0.885	0.853
**MMseqs2**	0.941	0.926	0.898	0.874	0.84

#: The lowest value of *T* allowed for CD-HIT is 0.8

*: Only CD-HIT, UCLUST, and MMseqs2 can cluster protein sequences

Tables S6 and S7 show the silhouette coefficients of the sequence clusters derived by ALFATClust and other clustering tools respectively for the plasmid nucleotides datasets.

Table S6. Silhouette coefficients of sequence clusters derived by ALFATClust for the plasmid sequence datasets.

	γ_{low}		
	**0.8**	**0.75**	**0.7**
**Plasmid nucleotides**	0.723	0.716	0.727

Table S7. Silhouette coefficients of sequence clusters derived by other clustering tools for the plasmid sequence datasets.

	T^*				
	**0.9**	**0.85**	**0.8**	**0.75**	**0.7**
**CD-HIT**	0.627	0.654	0.665	N.A.#	N.A.#
**UCLUST**	0.693	0.697	0.676	0.705	0.686
**MMseqs2**	0.667	0.641	0.627	0.617	0.644
**VSEARCH**	0.698	0.698	0.697	0.716	0.694
**MeShClust**	-0.047	-0.061	-0.057	-0.065	-0.051

#: The lowest value of *T* allowed for CD-HIT is 0.8

*: DNACLUST cannot cluster some plasmid nucleotide sequences that are too short

References:

1. Li W, Jaroszewski L, Godzik A: **Clustering of highly homologous sequences to reduce the size of large protein databases**. *Bioinformatics* 2001, **17**(3):282-283.
2. Li W, Jaroszewski L, Godzik A: **Sequence clustering strategies improve remote homology recognitions while reducing search times**. *Protein Engineering, Design and Selection* 2002, **15**(8):643-649.
3. Steinegger M, Söding J: **Clustering huge protein sequence sets in linear time**. *Nature Communications* 2018, **9**(1):2542.
4. Ondov BD, Treangen TJ, Melsted P, Mallonee AB, Bergman NH, Koren S, Phillippy AM: **Mash: fast genome and metagenome distance estimation using MinHash**. *Genome Biology* 2016, **17**(1):132.
5. Baker DN, Langmead B: **Dashing: fast and accurate genomic distances with HyperLogLog**. *Genome Biology* 2019, **20**(1):265.
6. Fu L, Niu B, Zhu Z, Wu S, Li W: **CD-HIT: accelerated for clustering the next-generation sequencing data**. *Bioinformatics* 2012, **28**(23):3150-3152.
7. Li W, Godzik A: **Cd-hit: a fast program for clustering and comparing large sets of protein or nucleotide sequences**. *Bioinformatics* 2006, **22**(13):1658-1659.
8. Edgar RC: **Search and clustering orders of magnitude faster than BLAST**. *Bioinformatics* 2010, **26**(19):2460-2461.
9. Rognes T, Flouri T, Nichols B, Quince C, Mahé F: **VSEARCH: a versatile open source tool for metagenomics**. *PeerJ* 2016, **4**:e2584.
10. Ghodsi M, Liu B, Pop M: **DNACLUST: accurate and efficient clustering of phylogenetic marker genes**. *BMC Bioinformatics* 2011, **12**(1):271.
11. James BT, Luczak BB, Girgis HZ: **MeShClust: an intelligent tool for clustering DNA sequences**. *Nucleic Acids Research* 2018, **46**(14):e83-e83.
12. James BT, Girgis HZ: **MeShClust2: Application of alignment-free identity scores in clustering long DNA sequences**. *bioRxiv* 2018:451278.
13. Steinegger M, Söding J: **MMseqs2 enables sensitive protein sequence searching for the analysis of massive data sets**. *Nature Biotechnology* 2017, **35**:1026.
14. Chiu JKH, Ong RT-H: **ARGDIT: A Validation and Integration Toolkit for Antimicrobial Resistance Gene Databases**. *Bioinformatics* 2019, **35**(14):2466-2474.
15. Alcock BP, Raphenya AR, Lau TTY, Tsang KK, Bouchard M, Edalatmand A, Huynh W, Nguyen A-LV, Cheng AA, Liu S *et al*: **CARD 2020: antibiotic resistome surveillance with the comprehensive antibiotic resistance database**. *Nucleic Acids Research* 2019, **48**(D1):D517-D525.
